# Structural insights into cellobiose dehydrogenases of a non-lignocellulolytic fungus and their transcriptional dynamics across different nutritional modes

**DOI:** 10.1128/spectrum.02577-25

**Published:** 2025-12-31

**Authors:** Naike Kruhler, Sarbagya Ratna Shakya, Mukesh Dubey, Leander Sützl, Clemens Peterbauer, Magnus Karlsson, Mats Sandgren, Lea Atanasova

**Affiliations:** 1 Department of Molecular Sciences, Swedish University of Agricultural Sciences 8095, Uppsala, Sweden; 2 Department of Forest Mycology and Plant Pathology, Swedish University of Agricultural Sciences 469025, Uppsala, Sweden; 3 Department of Biotechnology and Food Science, BOKU University, Vienna, Austria; 4 Department of Agricultural Sciences, BOKU University, Tulln, Austria; Agroscope, Nyon, Switzerland

**Keywords:** CDH, AA3, transcriptomics, mycoparasite, structure function

## Abstract

*Clonostachys rosea* (*Hypocreales*, *Bionectriaceae*) is a ubiquitous fungus that colonizes living plants, digests organic material in soil (saprotroph) and parasitizes on or kills other fungi (necrotrophic mycoparasite) or nematodes and is thus of interest as a biological pest control agent. We recently found a high number of genes coding for cellobiose dehydrogenases (CDHs) in the *C. rosea* genome compared to significantly reduced or even missing gene content in saprotrophs such as *Neurospora crassa, Trichoderma reesei,* and other *Trichoderma* mycoparasites. Phylogenetic analysis of CDH enzymes in *C. rosea* revealed six members in AA3_1 class II and III, from which the enzymes of the class II group possess fungal carbohydrate-binding domains (CBM1). CDHs are extracellular enzymes produced by various wood-degrading fungi as they are proposed to be involved in cellulose, hemicellulose, and lignin biodegradation. Mass spectrometry analysis of class II CDHs confirmed the presence of oxidized products, consistent with the characteristics of single oxidized oligosaccharides, including glucose, mannose, maltose, cellobiose, lactose, and maltotriose. Structural and functional analysis revealed that class III *Cr*CDHs exhibit significant differences in active site composition when compared to class II enzymes; however, no activity was found on the tested substrates. The functional transcript analysis in the mycoparasite *C. rosea* revealed activation of all *cdh* genes during self-recognition, sensing, and contact with the host fungus. The differential expression analysis indicated specific involvement of these CDHs in mycoparasitism and wheat root colonization and in degradation of simple and complex cellulosic compounds such as microcrystalline cellulose and wheat straw.

**IMPORTANCE:**

This study provides the first characterization of the unusually enriched cellobiose dehydrogenase (CDH) gene family in *Clonostachys rosea*, a mycoparasitic fungus of interest for biological control. We found class-specific structural differences between multiple CDHs belonging to distinct phylogenetic classes. Transcriptome profiling demonstrates their differential activation during host sensing, mycoparasitism, and plant root colonization, suggesting novel ecological roles beyond classical lignocellulose degradation. These findings establish a pioneering framework for understanding CDH diversification and its contribution to different fungal lifestyles.

## INTRODUCTION

*Clonostachys rosea* (*Hypocreales*, *Bionectriaceae*) is a ubiquitous filamentous fungus that colonizes living plants, digests organic matter in the soil (saprotroph), and parasitizes or kills other fungi (necrotrophic mycoparasite) or nematodes (1–3) and is therefore of interest as a biological control agent (BCA). *C. rosea* has been shown to be an effective BCA against *Alternaria dauci* and *A. radicina* (4), *Sclerotinia sclerotiorum* (1), *Botrytis cinerea* (5), and *Fusarium* spp. (2), among others. Like prominent species of the mycoparasitic genus *Trichoderma*, *C. rosea* is not only an antagonist of other fungi but is also able to colonize and establish itself on living plant roots, demonstrating its rhizosphere competence (6), which can affect plant growth and defense system.

*C. rosea* strain IK726, used for whole- genome sequencing, was isolated from barley roots infected with *F. culmorum* and has been shown to be highly efficient as a BCA against a wide range of fungal and oomycete pathogens of agricultural and horticultural crops (7). We recently found a high number of genes coding for cellobiose dehydrogenases (CDHs) in the *C. rosea* genome compared to significantly reduced or even missing gene content in saprotrophs such as *Neurospora crassa, Trichoderma reesei,* and other *Trichoderma* mycoparasites. Phylogenetic analysis of CDH enzymes in *C. rosea* revealed six members in AA3_1 class II and III. This raises a central question: why does a non-lignocellulolytic fungus such as *C. rosea* retain and diversify a large CDH family? One possibility is that these enzymes have been repurposed beyond classical roles in lignocellulose degradation, potentially contributing to interactions with other fungi, host colonization, or adaptation to complex ecological niches.

CDHs (EC 1.1.99.18; AA3), which belong to the AA3_1 subfamily, are extracellular hemoflavoproteins and are an important component of fungal biochemistry (8, 9). They were initially identified in the white rot fungi *Trametes versicolor* and *Phanerochaete chrysosporium* during analysis of secretomes in the presence of cellulosic substrates (10). CDHs have since been recognized for their unique ability to oxidize cellobiose to cellobiono-1,5-lactone. CDHs are extracellular hemoflavoenzymes that play a critical role in lignocellulose degradation by generating reactive oxygen species and acting synergistically with other lignocellulolytic enzymes. These CDHs are composed of a flavin-containing dehydrogenase domain (DH) and a heme-containing cytochrome (Cyt) domain (9, 11), which enable an electron transfer process essential for efficient lignin and cellulose degradation. The Cyt domain, including its heme *b*, is also identified as a separate auxiliary activity (AA8 of iron reductases) in the CAZy database (8). A papain-sensitive flexible linker, approximately 20–35 amino acids in length, connects the two domains and provides significant mobility (12). This linker facilitates both open and closed conformations of CDH (11, 13). CDHs can reduce a variety of electron acceptors, including quinones, metal ions, and molecular oxygen, making them versatile biocatalysts in both natural and industrial processes. Their applications extend to biotechnological fields such as biofuel production, biosensor development, and bioremediation due to their ability to degrade complex polysaccharides and their potential use in enzymatic biofuel cells.

CDHs are classified into four classes: class I, class II, class III, and class IV (14). Based on phylogenetic analyses, basidiomycete CDHs are grouped in AA3_1 class I, whereas ascomycete CDHs are present in both AA3_1 class II and class III. The function of AA3_1 class III in ascomycetes remains largely uncharacterized, with the exception of *Fusarium solani Fs*CDH, which has been biochemically analyzed but has not yet been structurally characterized (14, 15). Class II CDHs are further subdivided into subclasses A and B based on the presence of a C-terminal type-1 carbohydrate-binding module (CBM1), which enables firm cellulose binding (14). Notably, class I and class IIB CDHs lack a CBM1; however, class I CDHs exhibit strong cellulose binding through a yet unknown mechanism (9).

CDHs have the ability to oxidize cellobiose, cellodextrins, and related oligosaccharides to produce cellobionolactone or cellobionate, accompanied by reduction of the flavin adenine dinucleotide (FAD) cofactor to FADH_2_ (16). Discriminating against monosaccharides, CDHs have been reported to have low catalytic efficiency toward glucose, galactose, and mannose (9). The carbohydrate substrate-binding site, located in the DH domain, is accessible through a 12-Å-long tunnel (17). CDHs complete their electron transfer chain by reducing terminal electron acceptors, including copper-dependent lytic polysaccharide monooxygenases (LPMOs) (18, 19). Reduction of metal ions at the Cyt domain could potentially lead to the generation of reactive hydroxyl radicals (OH·) via the Fenton reaction (20).

This study aims to unravel the functional and structural significance of multiple AA3 CDHs found in the biocontrol fungus *C. rosea*. Through a comprehensive analysis of the genetic, transcriptional, and proteomic landscapes, we also aimed to elucidate the differences of *C. rosea* CDHs in response to various nutritional cues. In addition, we investigated the detailed structural differences and enzymatic properties of individual CDHs to dissect the differences between the multiple enzymes in *C. rosea*. The findings from this study have not only enhanced our fundamental understanding of fungal ecology and biochemistry but also reveal novel members of the AA3_1 CAZy enzyme family, providing opportunities for further investigation of their potential biotechnological applications.

## RESULTS

### *C. rosea* possesses six *cdh* genes belonging to phylogenetically different subgroups of CDHs

**Fig 1:**
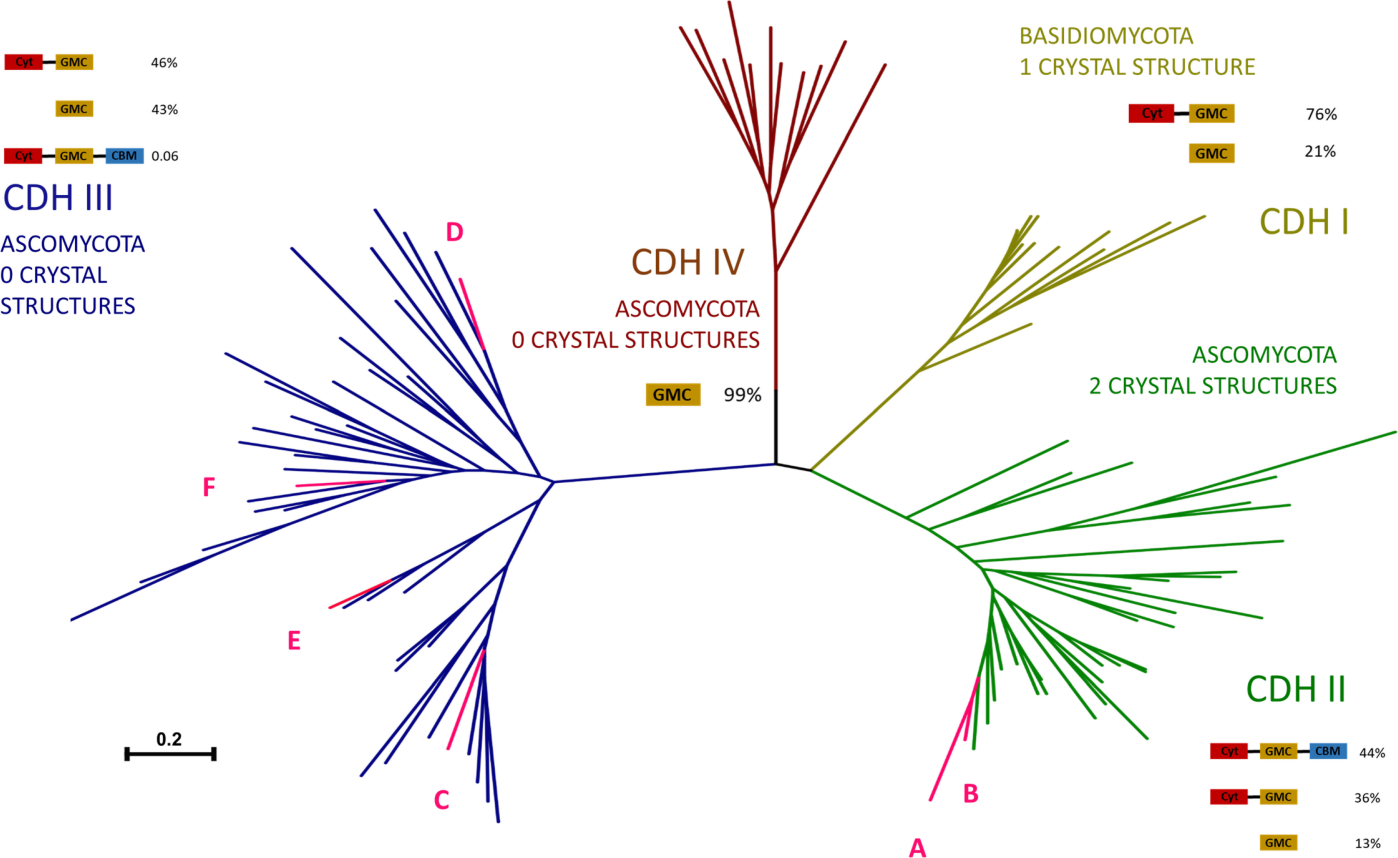
Phylogenetic analysis based on protein sequence selection of fungal CDHs from 14, representing the full extant sequence variation of this enzyme group. At each class, the percentage of domain architectures found within the class is shown (14), with DH (known also as glucose-methanol-choline [GMC] domain), Cyt, and CBM annotating for dehydrogenase, cytochrome, and CBM1, respectively. The species names and protein IDs of the included taxa are listed in Fig. S2. *Cr*CDH_A to E are abbreviated with capital letters A to E in the figure.

Our recent investigation revealed a considerable number of genes encoding CDHs in the *C. rosea* IK726 genome, a finding that contrasts with the significantly reduced or even absent gene content observed in saprotrophs such as *Neurospora crassa* and *Trichoderma reesei*, as well as in other *Trichoderma* mycoparasites (7). Phylogenetic analyses based on established fungal CDHs and *C. rosea* CDHs representing the full extant sequence variation of these groups of enzymes revealed that members of *C. rosea* CDHs are present in the clades of class II and class III groups (Fig. 1). The two *C. rosea* members of class II possess fungal CBM1 and exhibit high similarity to other sequences from this class. In contrast, the four class III members were identified within two statistically supported clades of class III (Fig. S2) and exhibited long C-terminal tails with an as yet unidentified function. The *Cr*CDH_C, which lacks a Cyt domain, and the *Cr*CDH_E were found to be more closely related than the *Cr*CDH_D and *Cr*CDH_F, which lack the conventional signal peptide sequence recognized by the SignalP. However, *C. rosea* class III CDHs did not show any duplication events and were rather highly diverged at the evolutionary level (Fig. 1).

### AlphaFold models highlight CDH class III variations in *Cr*CDHs

**Fig 2:**
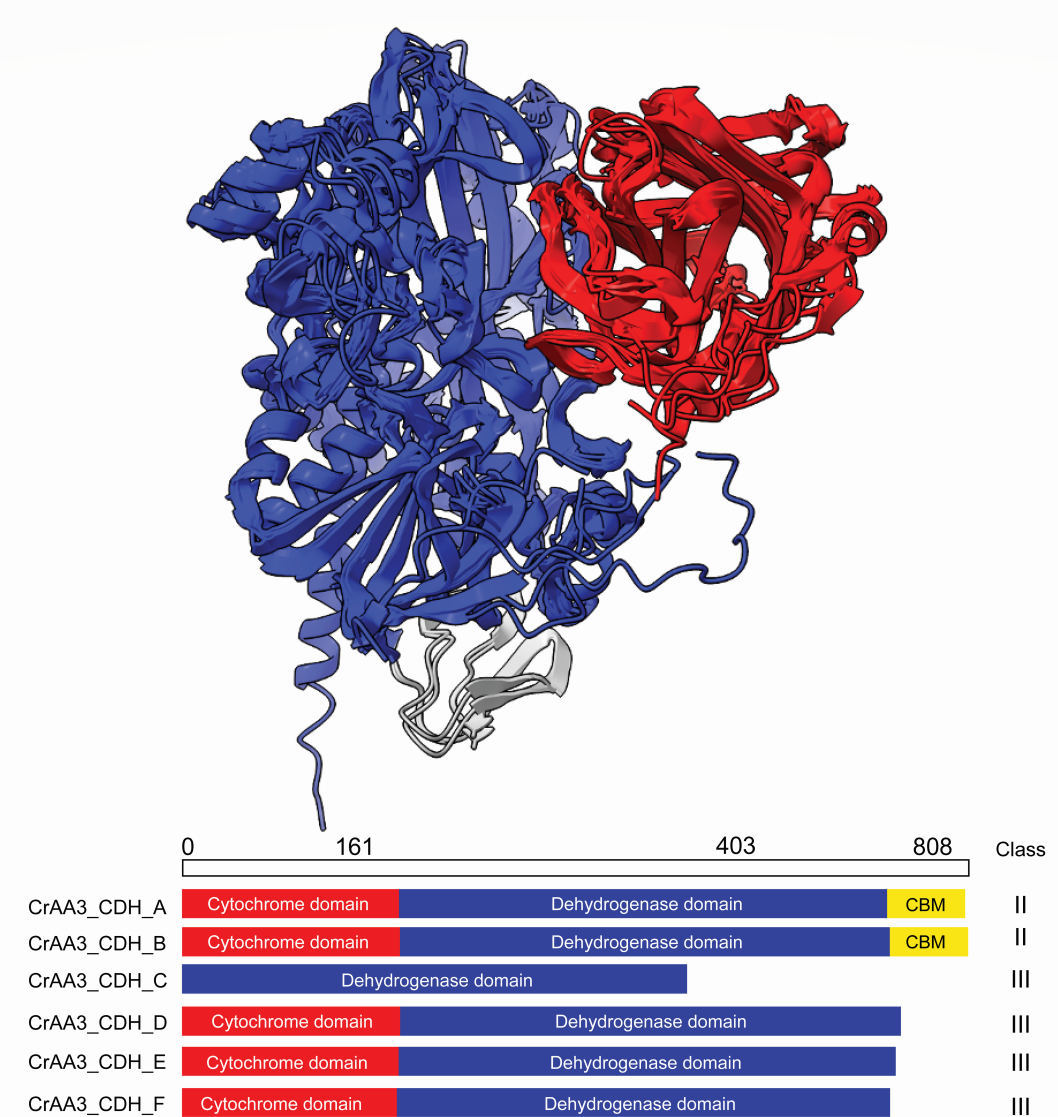
Overall comparison of *Cr*CDH_A to F structural domains. All enzymes contain a DH domain (blue). All CDHs except *Cr*CDH_C also possess a Cyt domain (red) connected to the DH domain. The carbohydrate module has been found only in the CDHs class II (*Cr*CDH_A and B). The sequence length of 804 amino acids corresponds to the *Cr*CDH_B with the longest sequence and is marked to indicate an approximate length and size of the domains.

The analysis of the six *Cr*CDH protein sequences revealed a close homology to the crystal structures of the *Neurospora crassa (Nc*CDH [PDB: 4qi6]) or *Myriococcum thermophilum (Mt*CDH [PDB: 4qi7]) proteins (11). *Mt*CDH was therefore used as a template for structure modeling of *Cr*CDH_D, *Cr*CDH_C, and *Cr*CDH_F with 35.5%, 38.1%, and 35.8% sequence identity, respectively. *Nc*CDH was used as a template for *Cr*CDH_A, *Cr*CDH_B, and *Cr*CDH_E with 55.1%, 62.3%, and 43% sequence identity, respectively. Comparison of all the *Cr*CDH AlphaFold structure models shows a high degree of structural similarity. All *Cr*CDHs consist of a DH domain containing the FAD and substrate-binding sites (21). Attached to the DH domain, *Cr*CDH_A and B have a C-terminal CBM1, which is not present in class III *Cr*CDHs. A notable feature of *Cr*CDH_C is that it does not have a Cyt-domain like all other *Cr*CDHs (Fig. 2).

**Fig 3:**
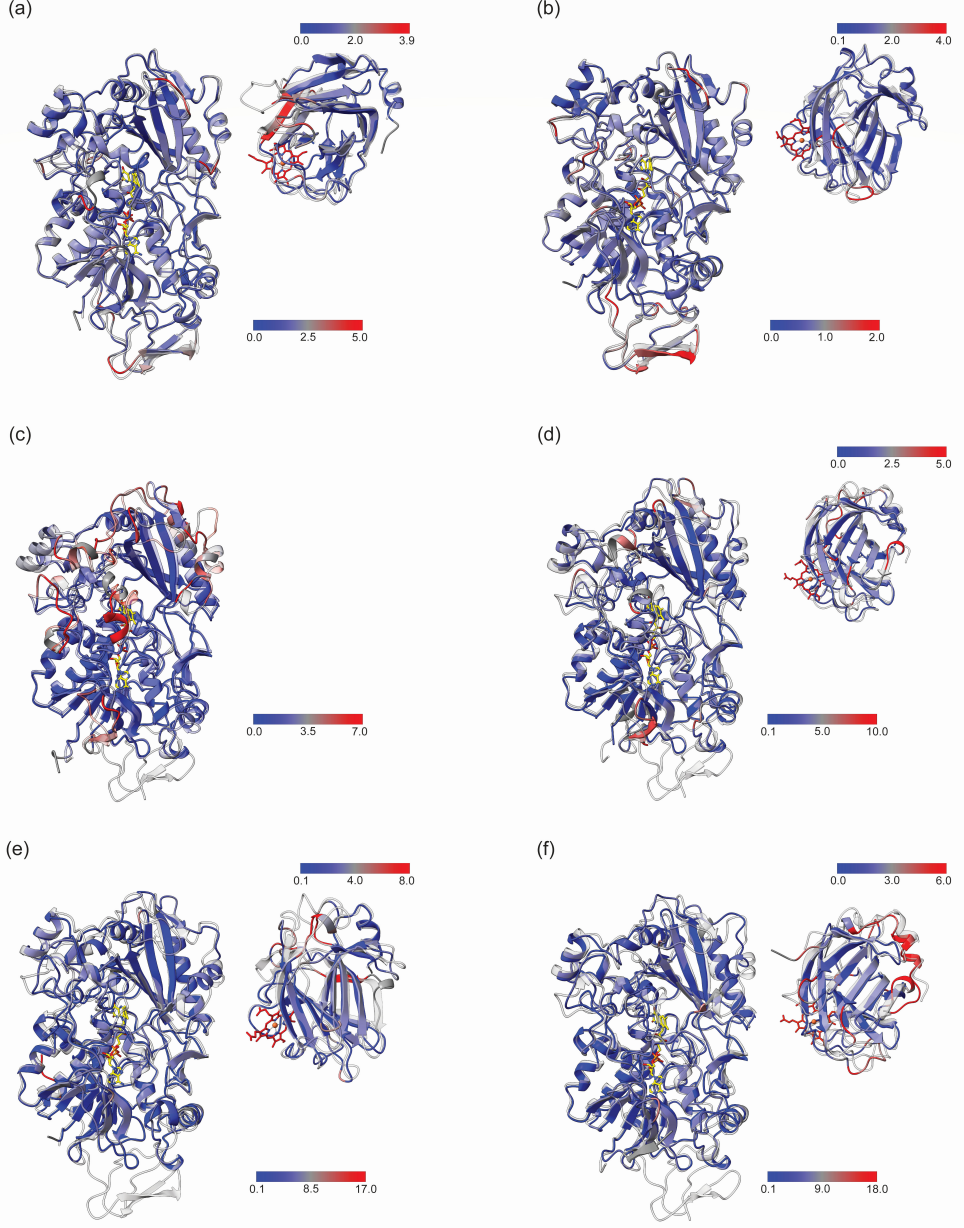
A root-mean-square deviation (RMSD) comparison of *Cr*CDH AlphaFold structures and the crystal structure templates *Mt*CDH and *Nc*CDH. The structures of the CDHs from *C. rosea* were calculated using the AlphaFold 2 algorithm. For each CDH, the closest homolog was selected as a template. In all cases, the DH and Cyt domains were employed as separate inputs and templates for structure modeling. The RMSD of the Cα backbone carbons of the *Cr*CDHs in comparison to their respective templates is represented by a color scale ranging from blue to red, indicating a low and high RMSD, respectively. The template is shown in gray and transparent. For each comparison, the RMSD scale is provided in the lower right-hand corner for the DH domain and in the upper right-hand corner for the Cyt domain. Residues for which no alignment could be achieved are indicated in gray. (**a and b**) The DH and Cyt-domains of *Cr*CDH_A and *Cr*CDH_B, respectively, compared with the template *Nc*CDH (PDB: 4qi6). (**c**) The DH domain of *Cr*CDH_C compared with its template *Mt*CDH (PDB: 4qi7). (**d**) The DH and Cyt domains of *Cr*CDH_D compared with its template *Mt*CDH (PDB: 4qi7). (**e**) The DH and Cyt domains of *Cr*CDH_E compared with its template *Nc*CDH (PDB: 4qi6). (**f**) The DH and Cyt domains of *Cr*CDH_E compared with its template *Mt*CDH (PDB: 4qi7).

A comparison between the AlphaFold models and the corresponding templates shows varying degrees of difference due to sequence similarity. *Cr*CDH_A, C, and E have a high sequence similarity, leading to similar folding patterns, whereas *Cr*CDH_C, D, and F have a low sequence similarity, leading to different folding patterns (Fig. 3).

*Cr*CDH_A and B are members of the CDH class II, as are *Mt*CDH and *Nc*CDH (11). These four enzymes have all three CDH domains (Fig. 2) (14). The CBM domains of *Cr*CDH_A and *Nc*CDH share high similarity (Fig. 3, blue color). However, this is not the case for the CBM domains of *Cr*CDH_B and *Nc*CDH, which have an RMSD of 2. In contrast, the DH domains of *Cr*CDH_A and B have a low RMSD compared to their templates, 0.484 and 0.411, respectively, with only a flexible loop at the substrate binding site, with RMSD values of 2 to 5. Looking at the structural models at this flexible region around the active site (marked with black lines in Fig. S19), the predicted Local Distance Difference Test scores are above 80% for all structures, apart from the region I in the *Cr*CDH_A, where the score drops to 60% (Fig. S19). The Cyt domain of *Cr*CDH_B shows a high degree of similarity, but a sheet near the heme-binding amino acids shows the highest dissimilarity compared to *Nc*CDH, possibly indicating a different mode of heme binding. The class III CDHs show the highest RMSD values compared to their template (Fig. 3).

*Cr*CDH_C exhibits the highest RMSD difference in the DH domain compared to *Mt*CDH (0.904), especially around the active site containing and binding the isoalloxazine of FAD where the substrate is catalyzed. In this domain, several helices and loops are shown in red in Fig. 3C, with RMSD values of 7, indicating a different function of this enzyme in *C. rosea* compared to the CDH class II enzymes. The total sequence identity of 39% indicates that the folding of *Cr*CDH_C differs from that of *Mt*CDH, what could potentially impact FAD binding. The absence of a CBM domain in *Cr*CDH_C and other class III *C. rosea* enzymes suggests the possibility of a distinct substrate specificity. The lack of a Cyt domain in the *Cr*CDH_C implies a divergence in electron transfer within the enzyme or to electron acceptors.

*Cr*CDH_D exhibits a relatively low similarity with its template *Mt*CDH at the catalytic center, as well as in the FAD-binding domain, particularly in the region where the CBM domain would be located. The DH domain of *Cr*CDH_E and F is highly similar to their template (Fig. 3; shown in blue), with an RMSD of 0 to 5. This indicates that the loss of the CBM domain does not affect the structure at the C-terminus. In contrast, in comparing the Cyt domain indicating a high RMSD (Fig. 3; e, f in red) to the Cyt domain of the class II template, up to an RMSD of 18 was observed.

These AlphaFold structures assist in elucidating the characteristics of CDHs from *C. rosea* in comparison to the publicly available CDH structures. Consequently, they facilitate the identification of potential targets for further characterization of novel CDHs belonging to class II or class III, the latter of which has not yet been structurally characterized (15).

A comparative analysis of the CDH structures reveals a high degree of similarity, with only minor variations in the amino acid sequence. This underscores the necessity for further investigation into the active sites of these enzymes.

**Fig 4:**
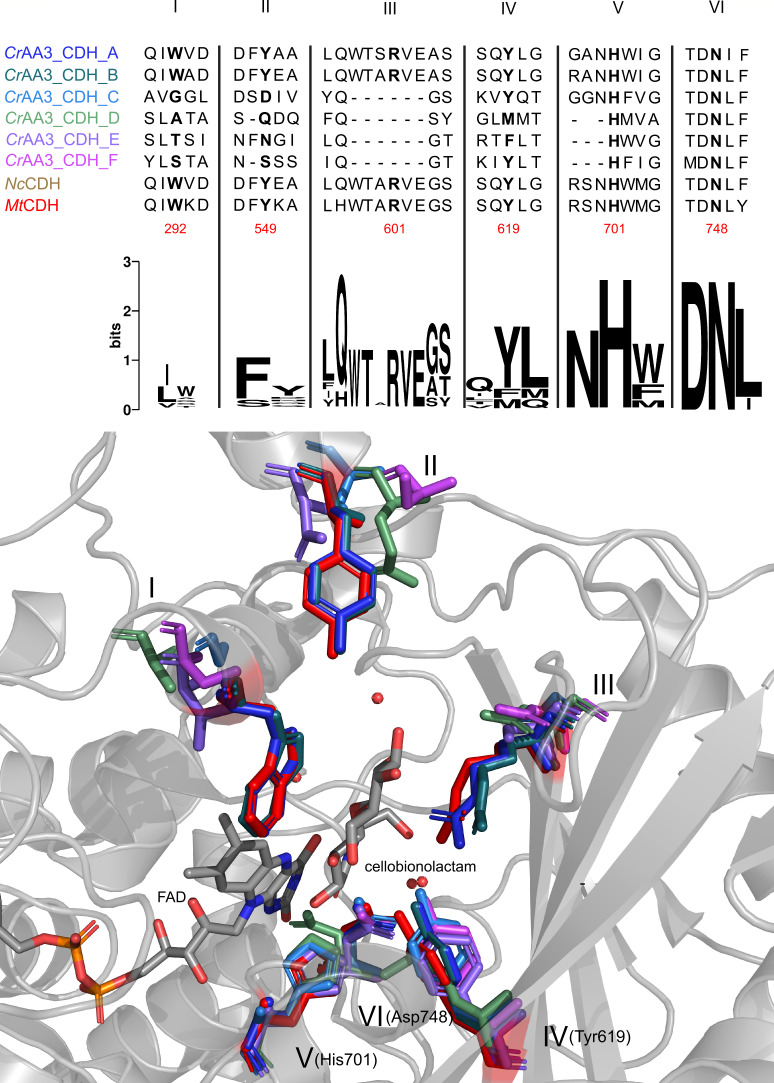
A comparative analysis of the predicted *Cr*CDH AlphaFold structures and the *Mt*CDH crystal structure based on a sequence alignment containing the proposed catalytic active residues for *Cr*CDH_A and *Cr*CDH_B, *Nc*CDH (PDB: 4qi6), and *Mt*CDH (PDB:4qi5). The alignment areas are numbered from I to VI, with the key amino acids highlighted in bold. The amino acid position for *Mt*CDH is indicated in red below the alignment boxes. Tyr619, Asp748, and His701 from *Mt*CDH discussed in the text are additionally annotated in the figure. Sequence logos representing the catalytically active amino acids and their neighboring residues are shown from I to VI. *Mt*CDH is depicted in gray, with the amino acids represented as sticks shown in red. The essential amino acids of *Cr*CDH are color-coded to match the corresponding name color in the alignment on top. The active site residues are labeled from I to IV, aligning with the sequence numbering indicated above the sequence alignments.

The AlphaFold-predicted structure models demonstrate a comparable arrangement of amino acids within the active site that are responsible for substrate binding and catalysis (Fig. 4). The structure of *Mt*CDH was determined at 2.4 Å with its inhibitor, cellobionolactam, utilized as a comparative template (11).

The His701 at position V and Asp748 at position VI are known to catalyze the reaction of the substrate and oxidize the sugar with the co-factor FAD, either at the C1 or C4 position. These residues are highly conserved among all CDHs (17).

Tyr619 at position IV of *Mt*CDH is responsible for stabilizing the transition state during cellobiose oxidation, as evidenced by mutation studies (11). However, in *Cr*CDH_D and E, the amino acids at the equivalent positions are either a Met or Phe. Both amino acids are nonpolar and possess hydrophobic characteristics, which differ from those of Tyr619. In the same way, Phe could be employed in a comparable manner as it is well-suited for stacking interactions as an aromatic amino acid. In contrast, Met exhibits a distinct structural configuration, including a sulfur atom that may play a role in multiple intermolecular interactions.

The amino acids at position III exhibit the greatest degree of dissimilarity as the loop and the sheet are oriented in a wide range of configurations at this position. Following structural alignment, amino acids in close proximity to Arg601 were selected, demonstrating that *Cr*CDH belonging to class II exhibit identical amino acids at this position. However, this is not the case for *Cr*CDHs belonging to class III, where amino acids such as Ala (*Cr*CDH_C), Asp (*Cr*CDH_D), Ser (*Cr*CDH_E), and Asn (*Cr*CDH_F) are observed. One discrepancy is the length of the amino acid, due to which it does not extend deeply into the active site. Further examination revealed the presence of additional amino acids in the sheet to the right in the active site. *Cr*CDH_F, E, and C exhibit a similar conformation of the Arg or Lys residue, positioned in close proximity to the substrate consistent with structures of class II CDHs. In contrast, *Cr*CDH_D exhibits an Arg residue to the left of position III closer to the surface of the enzyme and a Phe to the left of position III. This differs from the shorter amino acids observed at this position in other CDHs, such as a Pro in *Mt*CDH and *Nc*CDH. Moreover, *Cr*CDH_C has an Arg to the right of the substrate binding area, which is positioned in a similar way to those in class II. In summary, the structural variability observed within the active site of *Cr*CDH enzymes, especially at regions I, II, and III, highlights distinct differences between class II and class III CDHs. Notably, region III exhibits considerable variability in the loop and sheet orientations, with class II CDHs displaying conserved amino acids near Arg601, while class III *Cr*CDHs show a greater diversity in residues, such as Ala, Asp, Ser, and Asn. This suggests that this region is important for the divergence in substrate recognition or binding stability in this group of enzymes. Similar discrepancies are also seen in region II, where class III *Cr*CDHs harbor distinct residues compared to class II enzymes. Additionally, the aromatic residues in region I of class III CDHs, which differ from the Trp in class II, further emphasize the differences in the substrate binding and cofactor interactions. These structural distinctions suggest that class III *Cr*CDHs may interact with their substrates in a fundamentally different manner compared to the class II enzymes, potentially leading to alternative catalytic reaction mechanisms for class III enzymes.

### *C. rosea cdh* genes are differentially regulated during different nutritional modes of this fungus

**Fig 5:**
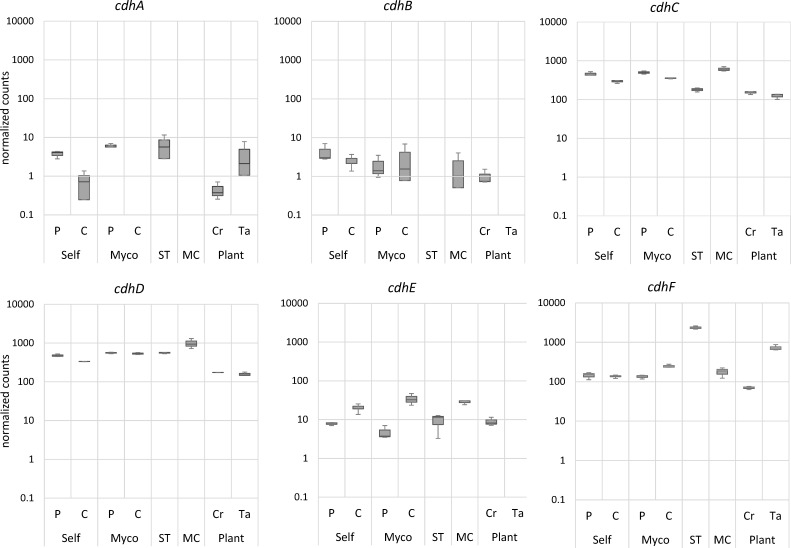
Expression levels of *C. rosea cdh* genes under different nutritional conditions, shown as DESeq2 size factor–normalized transcript counts. Conditions include sensing of itself (Self P) and of the host fungus *Fusarium culmorum* (Myco P), self-recognition during direct contact with itself (Self C) or the host fungus (Myco C), growth on MC or ST, and colonization of wheat roots (Plant Ta) compared to control conditions without the plant (Plant Cr).

**Fig 6:**
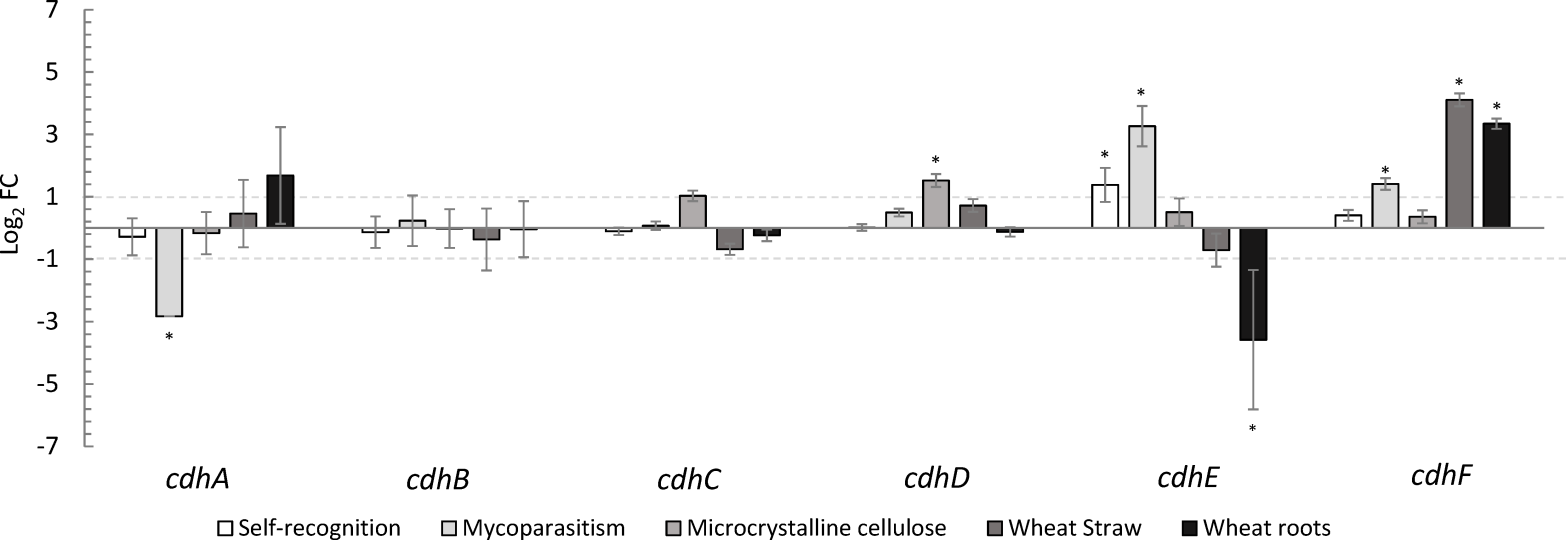
Differential gene expression analysis of *C. rosea cdh* genes under different conditions revealed their involvement in self-recognition, mycoparasitism, cellulose and wheat straw degradation, and interaction with wheat roots. Bars represent log₂ fold changes with standard errors. Asterisks indicate statistically significant differential expression (*P*-value and adjusted *P*-value < 0.05, DESeq2 analysis).

We performed a transcriptional analysis of *C. rosea cdh* genes to gain insights into their specific roles and regulatory mechanisms in this fungus. To gain a deeper understanding of the involvement of CDH in the nutritional strategies of this fungus (Fig. 5), we analyzed the transcriptional levels of these enzymes in self-recognition, saprotrophy (microcrystalline cellulose [MC] and wheat straw [ST]), mycotrophy (host fungus *F. culmorum*), and direct plant interaction (wheat roots). Functional transcript analysis revealed activation of all *cdh* genes during self-recognition, sensing, and contact with the host fungus. The *cdhA* and *cdhB*, which belong to the class II AA3_1 enzymes, showed a rather moderate transcriptional response. *CdhA* was expressed during the sensing phase (pre-contact) toward itself and the host (Fig. 5; Self P and Myco P, respectively); however, it was not transcribed when *C. rosea* started to interact with the host fungus (Fig. 5; Myco C). Interestingly, this gene was also not transcribed on MC under the given experimental conditions but was upregulated when *C. rosea* colonized living wheat roots (Fig. 6). In contrast, *cdh*B, which also belongs to class II AA3_1 typically active on cellulose, was not transcribed on wheat straw and in contact with wheat roots. Nevertheless, a proteomic study of *C. rosea* grown in liquid culture supplemented with MC showed a time-dependent expression of both class II CDH enzymes as early as day 2, peaking on day 10 of cultivation (Table 1). The differences in transcription and time-dependent expression can be explained by differences in the substrate availability in the different cultivation methods, as well as by the observation that these are both differentially functional genes.

**TABLE 1:** List of *C. rosea* CDHs secreted on microcrystalline cellulose on the second, fifth, and tenth day of cultivation^*a*^

CDH	E-value	Sequence coverage [%]	Unique peptides	MW [kDa]	Time [days]
*Cr*CDH_A	−6.2	2.8	2	88.6	2
	−132.1	21	15		5
	−307.4	39	29		10
*Cr*CDH_B	−89.5	16	12	88.6	5
	−201.6	28	19		10

^*a*^ The E-value represents the base-10 logarithm of the expectation that a given peptide assignment was made at random, while the coverage [in %] annotates the sequence covered by the identified peptides. The number of distinct peptide sequences found in the protein group, number of spectra that were matched to any particular protein in every sample, the calculated molecular weight (MW), and the sampling time points at which the proteins were detected are listed.

Class III genes *cdhC*, *cdhD,* and *cdhF* were found to be highly transcribed in all conditions, with *cdhC* and *D* showing the highest transcription on MC and *cdhF* being highly transcribed on ST and when in contact with live plant roots (Fig. 5). In addition, *cdhF* was differentially upregulated more than fourfold and more than threefold compared to control conditions when *C. rosea* was grown on MC and during colonization of wheat roots, respectively (Fig. 6). *CdhE* was differentially upregulated during interaction with itself and with the host fungus *F. culmorum*, but was not transcribed during *C. rosea* colonization of wheat roots, leading to a significant downregulation of this gene during plant-fungal interaction (Fig. 6).

### *Cr*CDH_A and *Cr*CDH_B have the typical Class II substrate preferences and product formation

**Fig 7:**
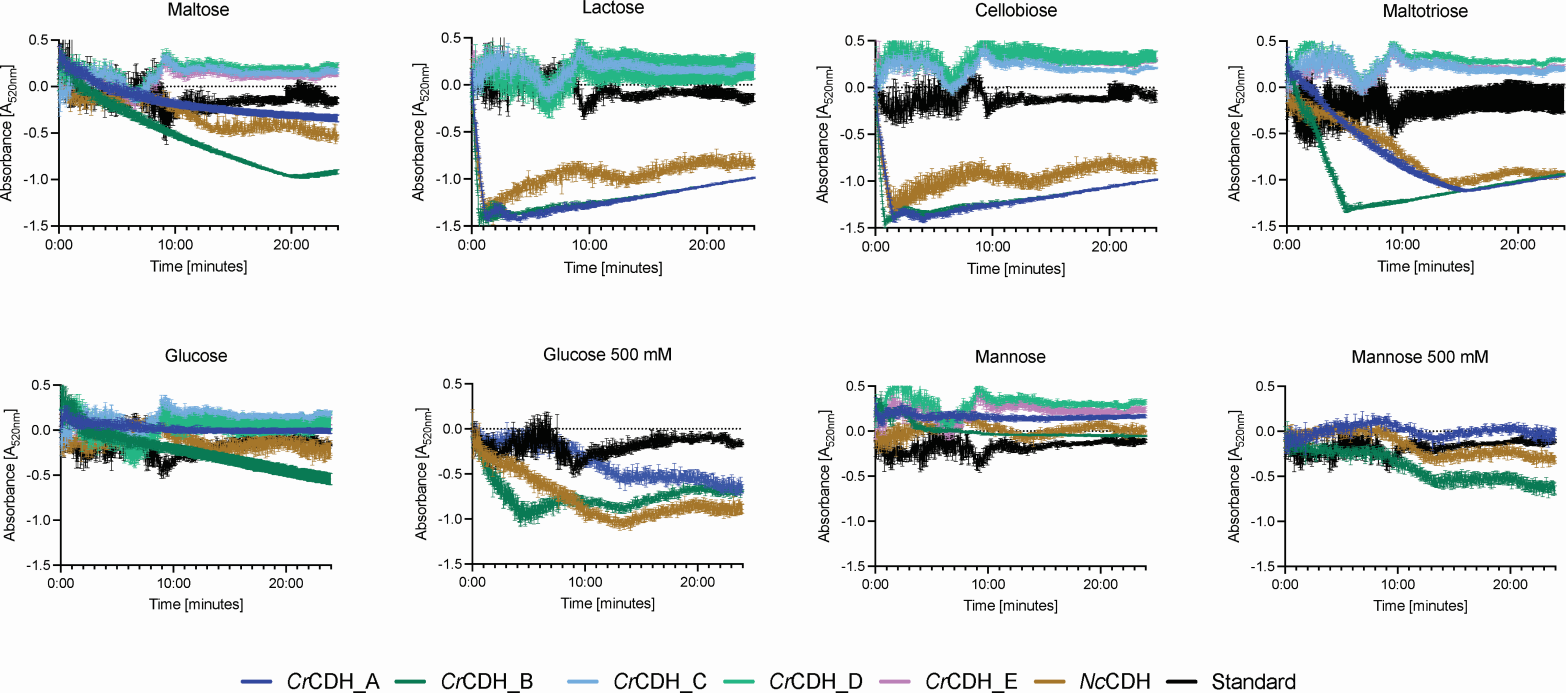
Relative activity of *C. rosea* CDHs on various substrates: cellobiose, lactose, maltose, maltotriose, glucose, and mannose. The change in absorbance, indicative of DCIP oxidation as an electron acceptor, was measured in the presence of C*r*CDH_A to E variants and N*c*CDH. Substrate concentrations were 50 mM, except for glucose and mannose, which were tested at 500 mM due to lower enzymatic activity. Measurements were taken over a 24-h period. The data were corrected by subtracting the absorbance of a standard containing the nontransformed *K. phaffii* supernatant as the enzymes were not purified. Values represent the mean ± standard deviation (SD) of triplicate samples, with error bars indicating the SD.

An initial screening of 95 substrates on FF Biolog plates demonstrated that *Cr*CDH_A and *Cr*CDH_B exhibited a specific preference for the monosaccharides glucose and mannose; the disaccharides cellobiose, lactose, and maltose; and the trisaccharide maltotriose (Table S2). *Cr*CDHs belonging to class III did not display any observable activity with any of the 95 carbon sources used in this investigation. The relative enzymatic activity of *Cr*CDHs was evaluated in triplicates using a 2,6-dichlorophenolindophenol (DCIP) assay, employing the specific substrates that demonstrated selectivity in the FF Biolog experiment (Fig. 7). Both class II *Cr*CDH_A and *Cr*CDH_B showed maximum relative activity to *Nc*CDH (also CDH class II) on cellobiose (0.009 U/mL, 0.02 U/mL, and 0.01 U/mL, respectively) and lactose (0.014 U/mL, 0.023 U/mL, and 0.013 U/mL, respectively). Further, *Cr*CDH_B demonstrated elevated volumetric activity with 0.01 U/mL on maltose and 0.006 U/mL on maltotriose compared to *Nc*CDH (0.002 U/mL). Notably, both *Cr*CDHs from class II and *Nc*CDH exhibited low activity toward disaccharide maltose (0.0002 U/mL, 0.0006 U/mL, and 0.0003 U/mL), which contains alpha 1,4-glycosidic links as already reported in 22, 23. This suggests that the active site of CDHs may be selective for beta 1,4-glycosidic bonds. Comparing all six tested substrates (Fig. 7), the monosaccharides, 50 mM glucose and mannose, exhibited the lowest activation of all tested enzymes. The enzymes displayed a lack of activity with regard to mannose, exhibiting no effect at lower sugar concentrations. However, when the concentration was increased tenfold, a considerable amount of activity was observed with glucose, which was the case for *Cr*CDH_A (0.0003 U/mL), *Cr*CDH_B (0.003 U/mL), and *Nc*CDH (0.001 U/mL). It is noteworthy that when the substrate concentration was increased, *Cr*CDH_B and *Nc*CDH showed activity on mannose (both 0.0002 U/mL), while *Cr*CDH_A remained inactive.

**Fig 8:**
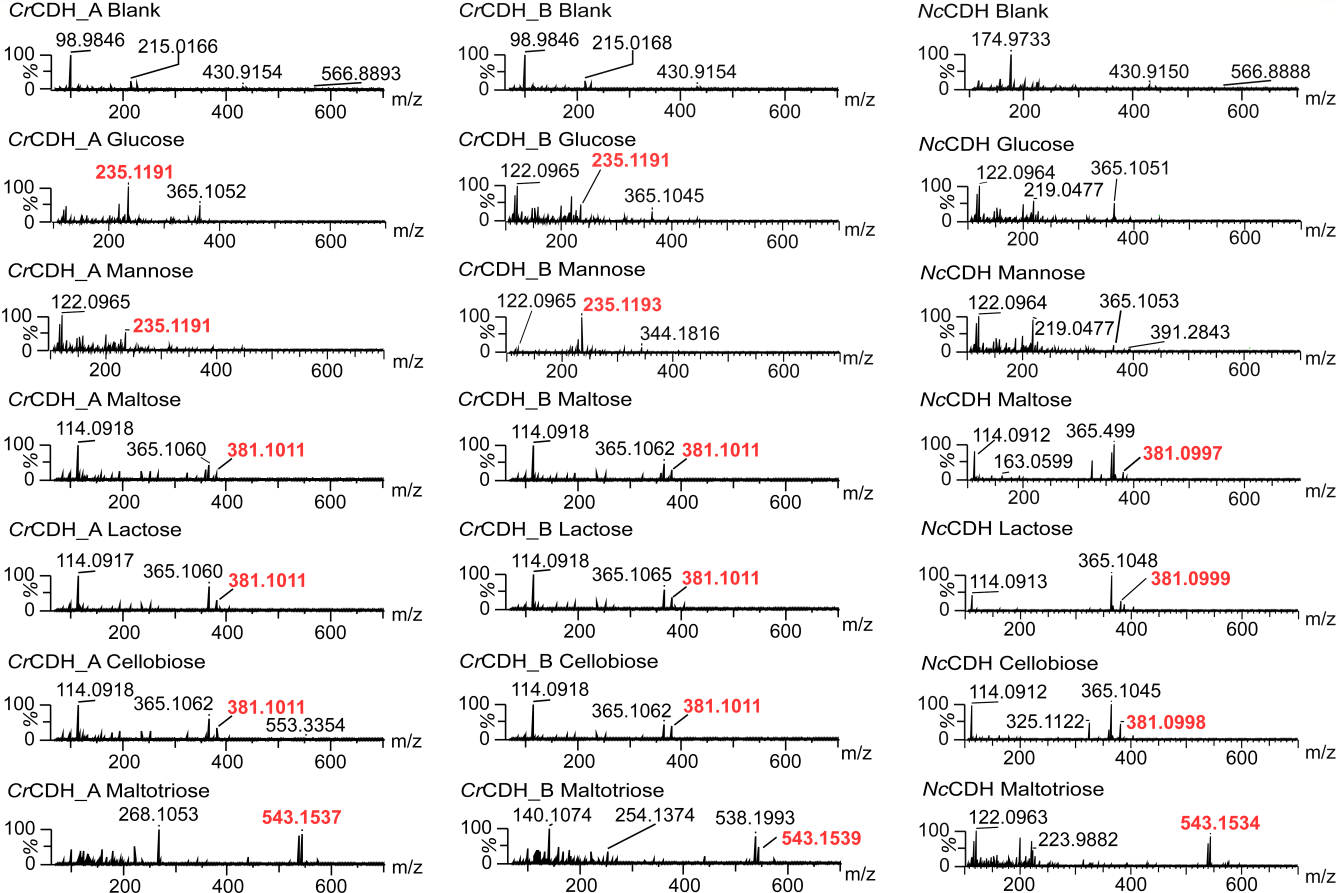
Positive MS spectra of standard sugars tested for activity. The spectra show the masses of the products as sodium adducts detected approx. at +1 and −1 min before and after the respective sugar’s glucose (∼6.78 min, 235 *m*/*z*), mannose (∼6.79 min, 235 *m*/*z*), maltose (∼7.95 min, 381 *m*/*z*), lactose (∼7.95 min, 381 *m*/*z*), cellobiose (∼8.37 min, 381 *m*/*z*), and maltotriose (∼8.98 min, 543 *m*/*z*) eluted from the liquid chromatography column. For the protein control, no time was selected, and the mass spectra of the whole chromatogram are shown. The controls for the sugars as substrates are shown in Fig. S4 to S6. The sugars were detected as sodium adducts. The samples were taken from the activity assay plate and contained buffer, DCIP additionally to the sugar and respective enzyme supernatant and were incubated for 24 h, before filtering with a 10 kDa filter and injected into the LC-MS. The positive control of N*c*CDH proved that the activity assay and the detection method of the substrates and products work, and product formation by C*r*CDH_A and B can be detected.

Furthermore, the 24 h supernatant assays were filtered (10 kDa) and analyzed using ESI-Orbitrap-MS to validate the activity obtained using the supernatant of the *K. phaffii* X33 strain as a control and the supernatant of the X33 overexpression of *Cr*CDH_A and B. Only samples that exhibited positive results in the DCIP activity assays were subjected to further analysis using mass spectrometry (MS) (Fig. 8). Moreover, the assays were conducted with *Nc*CDH as a positive control to demonstrate the efficacy of the methodology. The objective of this analysis was to verify substrate turnover, rather than to quantify or characterize the specific activity against the carbohydrates tested. Both *Cr*CDH_A and *Cr*CDH_B exhibited activity toward the same carbohydrates tested, glucose, mannose, maltose, cellobiose, lactose, and maltotriose, in the DCIP assay. The analysis of the activity assay in the MS-positive mode revealed that the carbohydrates exhibited a positive charge [M-H]+, with an increase in mass of one sodium molecule of ~22.99 Da. The oxidation of the substrate resulted in an increase in mass of ~16 Da. It should be noted that no further analysis was conducted afterward; therefore, it is unclear if the oxidation took place at the C1- or C4-position of the sugar. Consequently, the exact position of oxidation cannot be determined, being either the corresponding acid (e.g. cellobionic acid, if the oxidation is taking place at the C1-position), or the C4 gemdiol/keto group, also known as gemdiol/keto-equilibrium oxidation (24, 25).

A considerable amount of background noise was observed in the supernatant of the control *K. phaffii* X33 (Fig. S6). This background noise was taken into account during the evaluation of the obtained data. This observation was made for all samples containing enzymes, including *K. phaffii* X33, *Cr*CDH_A, and *Cr*CDH_B.

The detection of glucose was confirmed at 203 *m/z* with a retention time of 4.5 min in the control sample and all other samples. Upon addition of *Cr*CDH_A and B, the detection of oxidized glucose at 235 *m/z* with a retention time of 6.8 min was also observed, though at a very low concentration in the analyzed sample. Oxidized glucose was not detected for *Nc*CDH (Fig. 8), despite the observation of enzyme activity in the DCIP assay.

Mannose was identified at the identical mass-to-charge ratio and retention time as that of the substrate and oxidized product of glucose. The observed activity was identical to that observed with glucose, indicating that these enzymes exhibit low activity against monosaccharides. This finding was previously demonstrated for *Nc*CDH (26).

Additionally, the product of glucose was identified in the *K. phaffii* X33 control sample, though in very low quantities (Fig. S8). These levels were markedly lower than those observed for *Cr*CDH_A and B, where a distinct peak was evident in the chromatogram and a relative abundance of 100% was observed at identical retention time points (Fig. S10 and S13).

The results for mannose are identical to those for glucose. Oxidized mannose in the *K. phaffii* X33 control (Fig. S8) is also present, but once more, *Cr*CDH_A (60% relative abundance) and *Cr*CDH_B (100% relative abundance) contain considerably higher concentrations of oxidized mannose (Fig. S12 and S15). Regarding *Nc*CDH, no product formation could be detected for the monosaccharide mannose (Fig. S18).

The retention time points for cellobiose, maltose, and lactose were observed to range from 7 to 8.5 min, while the substrate exhibited a mass-to-charge ratio of 361 *m/z*, as a sodium adduct.

The oxidation of the disaccharides resulted in the detection of a mass with a value of 381, which was observed with all enzymes, including *Cr*CDH_A and B, as well as *Nc*CDH. The absence of a product in the standard and *K. phaffii* X33 control indicates that only the enzymes are responsible for this oxidation of the substrate (Fig. S9). The substrate and product eluted in close proximity to one another, yet a product peak of nearly equivalent intensity to that of the substrate was observed in the chromatogram for *Cr*CDH_A and B with cellobiose, marking the highest activity in all assays.

The single oxidation of the trisaccharide maltotriose was confirmed by the formation of the oxidized product with a mass-to-charge ratio of 543 *m/z*. However, these results require further examination as the product was also detected in notable concentrations in the standard and the *K. phaffii* X33 control (Fig. S9). The detection of the product in these samples suggests that the sugar contained impurities or that spontaneous oxidation of the substrate had occurred. It may be postulated that the product formation in *Cr*CDH_A (relative abundance 100%) and *Cr*CDH_B (relative abundance 55%) is greater than that observed in the standard, which exhibited a relative abundance of 50%. However, as the precise enzyme concentrations are unknown, the results with maltotriose must be validated using pure enzyme and pure sugar samples, as well as with *Nc*CDH, which exhibited the lowest maltotriose activity (Fig. S12, S15 and S18). The results show that both *Cr*CDH_A and *Cr*CDH_B exhibit the same activity found for previously characterized CDH class II members, and *Cr*CDH_A and *Cr*CDH_B are thus both correctly classified within this AA3 subgroup.

## DISCUSSION

CDH enzymes are members of the CAZy AA3_1 subfamily and the GMC oxidoreductase superfamily, which is a family of flavoproteins with oxidoreductase activity. CDHs are classified into four distinct AA3 classes based on phylogenetic analyses (14): class I–IV. Class I CDHs are exclusively found in basidiomycetes, while ascomycetes harbor class II, class III, and class IV CDHs. While class II contains several well-characterized CDHs, the class III CDHs remain poorly characterized. Currently, the only available biochemical characterization of class III CDHs is of *Fs*CDH, showing that this enzyme is active against typical CDH substrates such as cellobiose and lactose, and with electron acceptors, resembling class I and II CDHs. This finding raises questions about the evolutionary role of the abundant class III CDHs in fungi. Further studies comparing CDH classes will be key to uncovering their biological functions and evolutionary significance (15). Class II CDHs is further subdivided into two subclasses: A and B. This subdivision is based on the presence (A) or absence (B) of a CBM1, which enables strong cellulose binding (14). This classification system highlights the structural and functional diversity of CDHs across different fungal groups, suggesting potential adaptations to various ecological niches and substrates. The abundance and diversity of CDH genes in *C. rosea* IK726 present an intriguing contrast to other fungal species, particularly saprotrophs and mycoparasites that possess smaller arsenal of these genes. This genetic abundance of CDHs suggests a functional significance in the lifestyle of *C. rosea*. The phylogenetic distribution of *C. rosea* CDHs across both class II and class III of the AA3_1 subfamily, along with their structural variations, indicates a complex evolutionary history. The presence of unique features, such as the long C-terminal tails in class III CDHs and the absence of conventional signal peptides in some members, raises questions about their specific roles and adaptations. Furthermore, the high divergence observed in class III CDHs, without evidence of recent duplication events, points to long-term evolutionary processes shaping these enzymes.

Structural studies of AA3 enzymes carried out so far indicate that these proteins are composed of an N-terminal FAD-binding domain and a C-terminal substrate-binding domain. The FAD-binding domain forms the alpha-beta fold typical of dinucleotide binding proteins, whereas the substrate-binding domain comprises a beta sheet surrounded by alpha helices. The general topology of these proteins is conserved, although inserted structural elements are present in both choline dehydrogenases and alcohol dehydrogenases. In accordance with the classification system established by the CAZy database (8), the AA3_1 subfamily includes CDHs (EC 1.1.99.18), which are extracellularly produced by a multitude of lignocellulose-degrading fungi (9). It has been demonstrated that they are capable of generating hydroxyl radicals through Fenton-type reactions, which could potentially lead to the oxidation of lignin. In the existing literature, the core CDH was originally described as a monomeric protein with a bipartite domain organization comprising an N-terminal Cyt-domain of spectral class b connected to a C-terminal dehydrogenase domain containing the FAD redox cofactor (27, 28). These two distinct modules in CDH evolved in parallel as fused genes (29). Our results align with the previous finding showing that CDHs in *C. rosea* exhibit notable differences between class II and class III enzymes, at the active site. Class II CDHs *Cr*CDH_A and B are characterized by the presence of fungal CBM1, which facilitate their interaction with cellulose, suggesting a role in the degradation of plant biomass. In contrast, class III CDHs (*Cr*CDH_C, D, E, and F) lack these binding domains and instead possess long C-terminal tails, the functions of which remain unidentified. However, the overall structure and active site of CDHs class III are distinct from those observed in other CDHs. The absence of the CBM domain, which enables high specificity and binding to carbohydrates, and the absence of the Cyt domain, which is required for electron transfer, in *Cr*CDH_C give rise to questions regarding the function and activity of these enzymes. It is noteworthy that the Cyt domain appears to be highly conserved among all *Cr*CDHs, exhibiting a high degree of similarity to the AlphaFold template *Nc*CDH or *Mt*CDH. It has been demonstrated that the Cyt domain is crucial for the transfer of electrons to LPMOs (11). This raises the question of the appropriate electron acceptor for *Cr*CDH_C. Further analysis of *Cr*CDH_C may yield additional insights into the mechanisms of electron transfer and acceptor specificity in all CDHs. However, CDHs employ a wide spectrum of electron acceptors to oxidize soluble cellodextrins to the corresponding lactones (22). In addition to their role in LPMO activation, CDHs have been suggested to participate in quinone redox cycling, which plays a crucial role in lignin degradation. By reducing lignin-derived quinones, CDH could help prevent the repolymerization of lignin-derived radicals, thereby facilitating lignin depolymerization. This mechanism has been hypothesized for other flavin-dependent oxidoreductases, including pyranose 2-oxidase and aryl-alcohol oxidase, which interact with peroxidases and contribute to redox cycling in ligninolytic systems (30, 31). The potential involvement of *Cr*CDH_C in analogous processes makes further investigation necessary as it could provide insights into its physiological role beyond carbohydrate oxidation.

Nevertheless, despite using a class II CDH as a template, as no CDH class III crystal structure is so far available, the class III *Cr*CDH enzymes display a distinct active site configuration, suggesting differential activity or function of these enzymes. It is noteworthy that only two out of the six CDHs belong to class II, which has already been extensively characterized in the literature and also exhibits a highly conserved structure and active site between different species (11, 17). This is also the case for the class II *Cr*CDHs, which have the preferred structure for the oxidation of disaccharides (22). The AlphaFold (32) structural models of the *Cr*CDH offer initial insights into the variability of the *Cr*CDHs and the identification of distinctive activities within the class III CDHs. In particular, the active site of all structurally determined CDHs demonstrates that also other substrates could be bound, or the binding mechanism is different, as at position III (Fig. 4), a high variation of the amino acid type is found, which must result in a different mode of stabilizing the substrate binding or entrance. The polarity, hydrophobicity, and charge of the amino acids present at positions I, II, and III indicate that a different binding mode may be employed for substrates of an as-yet-unknown nature. The amino acids in these positions are either not present or located elsewhere in the sequence and possess distinct characteristics. For example, in place of Arg, an Lys is present (*Cr*CDH_C and E), or alternatively, the Arg is situated further to the left, closer to the surface, as in *Cr*CDH_D. With the exception of *Cr*CDH_D, all members of class III possess an Arg or Lys at position III in a different location. This is situated in close proximity to the position typically occupied by Arg601 in class II CDH. These discrepancies might have an effect on the binding of the second carbon ring. In particular, the Arg and Lys residues of *Cr*CDH_E, F, and C are situated in close proximity to the C1 position of the second ring of cellobiolactam. Furthermore, the Arg at position III of *Cr*CDH_C is situated to the right of other Arg observed in class II and is thus in a different position to the O3 of the second ring of cellobiolactam. It is notable that there are no amino acids with similar characteristics at these positions in the class II structures. At the conserved position IV, Tyr619 is responsible for stabilizing the transition state during cellobiose oxidation, as evidenced by mutation studies (11). However, in *Cr*CDH_D and E, the amino acids present are Met and Phe, both of which are nonpolar and possess hydrophobic characteristics, differing from those of Tyr.

The striking dissimilarities between CDHs belonging to class II and III, not only at position III but also at positions I, II, and IV, give rise to the question of which or how the substrates are bound in this active site. The variability observed in the regions responsible for substrate binding, such as positions I, II, and III, suggests that class III *Cr*CDHs could interact with different substrates, potentially with distinct binding modes or reaction pathways. In order to gain a deeper understanding of the different classes of CDHs and their catalytic activity, however, substrate specificities of *C. rosea* class III CDHs need to be discovered.

The activity of the class II *Cr*CDHs was successfully determined against a range of carbohydrates, including glucose, mannose, maltose, lactose, cellobiose, and maltotriose, through the use of activity assays utilizing DCIP as an electron acceptor and mass spectrometry (ESI-orbitrap-MS) analysis. The use of the *K. phaffii* X33 WT supernatant did not exert a considerable influence on the results. Previous studies have demonstrated that the discrimination against maltose and glucose is less pronounced in CDH class II, which aligns with the findings in other studies (22), as both *Cr*CDH_A and B exhibit low activities on these two monosaccharides.

Given that all *Cr*CDHs from class III are lacking a CBM domain and no activity for these has yet been detected, it can be speculated that these enzymes are unable to bind to cellulose or hemicellulose types of substrates and have been evolved to possess a different physiological role for *C. rosea*. This hypothesis has been previously demonstrated in studies of CDHs of class IIB (23). The positive control, *Nc*CDH, exhibited the expected activity, as has been reported previously (26), with the exception of its activity on glucose and mannose. The activity was detected by the DCIP assay, yet no product formation could be detected by MS, which gives rise to further questions and necessitates further activity characterizations. However, previously, a very low activity with a Km of 4,000,000 μM was detected for *Nc*CDH on glucose, but no supporting MS data were found in the literature (23).

The transcriptional analysis of CDH genes in *C. rosea* revealed a complex and context-dependent expression pattern, suggesting diverse roles for these enzymes in the fungus’s various ecological interactions. We examined CDH gene expression during self-recognition, saprotrophic growth on cellulose substrates, mycoparasitism, and during direct plant interaction. Class II CDH genes (*cdhA* and *cdhB*) showed moderate and specific expression patterns. The *cdhA* gene was shown to be transcribed during self-recognition and early host sensing on wheat straw and during wheat root colonization, but this gene was not transcribed on solid medium with addition of microcrystalline cellulose. Conversely, *cdhB* was transcribed on microcrystalline cellulose and relatively consistently during self and host interaction, but not on wheat straw or while colonizing wheat roots. Hoverever, he proteomic study of *C. rosea* in liquid culture supplemented with microcrystalline cellulose demonstrated time-dependent expression of both class II CDH enzymes (Table 1). The discrepancy between *cdhA* transcript detection on solid media and its corresponding protein presence in liquid culture may reflect distinct physiological responses to cultivation format. Liquid culture provides higher oxygen transfer and improved substrate accessibility, both of which can enhance secretion and stability of extracellular enzymes. In contrast, solid media often impose diffusion limitations and localized nutrient gradients, which may suppress transcription of certain genes while still allowing accumulation or persistence of secreted proteins. Such cultivation differences likely contribute to the observed variation in *cdhA* expression and secretion. Notably, the transcript counts for these two class II *Cr*CDHs were low compared to the overall counted transcripts in class III, suggesting that the class II CDHs are specifically, but efficiently expressed in *C. rosea*. Our structural and activity analysis of *Cr*CDH_A and B demonstrated their activity on cellulose-like materials.

Furthermore, class III *Cr*CDHs did not show any detectable substrate activity on tested carbon sources; however, class III *Cr*CDH genes (*cdhC* to *cdhF*) demonstrated high transcription levels across various ecological conditions. The absence of detectable catalytic activity suggests that class III enzymes may serve alternative biological roles. They might contribute to redox-based sensing or signaling processes that help coordinate responses during fungal-fungal or fungal-plant interactions. Another possibility is that they function through interactions with other secreted proteins, indirectly shaping the extracellular environment rather than catalyzing substrate oxidation. The genes *cdhC* and *cdhD* were particularly transcribed on microcrystalline cellulose, while *cdhF* showed high expression on wheat straw and during root colonization. The downregulation of CDH genes during plant root colonization aligns with previous findings, where *cdhB*, *cdhD*, *cdhE*, and *cdhF* were reported to be significantly downregulated in *C. rosea* during its interaction with wheat roots seven days post inoculation (33). This distinct expression profile indicates a specialized role for *cdhE* in fungal-fungal interactions. The varied expression patterns observed across different growth conditions and interaction scenarios underscore the functional diversity within the CDH gene family in *C. rosea*. These findings suggest that CDHs play multifaceted roles in the adaptive strategies of *C. rosea*, contributing to its success as a versatile mycoparasite and plant-associated fungus. Traditionally, CDHs have been characterized as extracellular oxidoreductases primarily involved in cellulose degradation, where they oxidize cellobiose and related oligosaccharides and transfer electrons to lignocellulolytic systems such as LPMOs in wood-decaying and saprotrophic fungi. However, our findings in *C. rosea*, a nonlignocellulolytic mycoparasite, challenge this view: multiple CDH genes are expressed under conditions unrelated to cellulose degradation, including fungal-fungal and fungal-plant interactions. Such expression patterns suggest that CDHs in *C. rosea* may have been evolutionarily repurposed toward alternative redox or signaling functions rather than classical carbohydrate oxidation. Future biochemical and structure-function studies will be essential to determine the true substrate specificities and biological roles of these enzymes.

This study highlights the importance of considering environmental context when interpreting gene function and emphasizes the need for further research to elucidate the specific roles of each CDH enzyme within the complex ecological environments of *C. rosea*.

## MATERIALS AND METHODS

### Cultivation, cloning, and transformation of microorganisms

The transcripts for the expressions of the six *C. rosea* IK726 CDH enzyme, *cdhA*, *cdhB, cdhC, cdhD, cdhE,* and *cdhF,* genes corresponding to the gene numbers CRV2G00004950, CRV2G00016484, CRV2G00006724, CRV2G00010087, CRV2G00016243, and CRV2G00018742, respectively,were commercially synthesized (BioCat, Germany) and subcloned into the commercial pPICZA vector (Thermo Fisher Scientific, Germany) containing its native signal peptide sequences. The genome of the strain IK726 was sequenced and is publicly available (7). The inserted genes were expressed under the regulation of the pAOX1 and AOX1tt. Plasmids were linearized with *Sac*I restriction enzyme and were transformed to *Komagataella phaffii* using an established protocol. Briefly, linearized DNA (~100 ng) was added to electro-competent *K. phaffii* X33 cells, and the mixture was electroporated (1.5 kV, 125 Ω, 3 ms) and recovered by addition of yeast extract peptone dextrose (YPD) medium and incubated for 4 h at 300 rpm at 30°C. The cells were then plated to YPD agar plates with 100 µg/mL zeocin (Invitrogen, Germany). Colonies were picked from the plate and were tested by colony polymerase chain reaction (PCR) to confirm the integration of the plasmid. Each picked colony was mixed in 20 µL of 0.02 M NaOH and heated at 100°C for 10 min and was then used as a template for the PCR. Colony PCRs were performed using the primers 5′AoX_fw GACTGGTTCCAATTGACAAGC and 3′AoX_rv GCAAATGGCATTCTGACATCC, targeting the AoX1 promoter and terminator. These inserts were cut, purified, and confirmed by sequencing.

### Expression of AA3_1 CDHs in *K. phaffii*

The *K. phaffii-*transformed cells were cultivated in YPG plates containing 500 mg/mL zeocin (Invitrogen, Germany) media for 3 days at 25°C. The selected colonies were used for the cultivation following the methanol feeding strategy described in Invitrogen’s *Pichia* Fermentation Guidelines (34). For each *K. phaffii-*expressed CDH enzyme, one colony was inoculated into technical triplicates of 100 mL YPG medium in 250 mL baffled flasks and incubated for 2 days at both 15°C and 28°C. On the third day, the cells were harvested by centrifuging the cultures at 4,000 × *g* and 4°C for 10 min. The harvested cells were re-suspended with 100 mL of YP media and mixed thoroughly. The media was then divided into three equal proportions in 250 mL baffled flasks, and the induction phase was started for 6 days at 15°C or 28°C at pH 5.5, respectively (Fig. S1). The induction of protein expression was started by adding 0.5% of methanol of the total culture volume at 0 h of cultivation. After 24 h cultivation, the induction interval decreased to 12 h for the next 5 days, using 1% of methanol. The cultivation media were harvested after 6 days and cultivated by centrifugation at 4,000 × *g* for 10 min at 4°C. The resulting supernatants were filter-sterilized gradually by using 0.47, 0.45, and 0.2 µm filters. The sterilized culture filtrates were then concentrated using Vivaflow 200 concentrator with an MW cut-off of 10 kDa to obtain a final concentrated volume of 20 mL. The expression of the CDHs was visualized and verified using SDS gels (BioRad, Mini-Protean TGX stain free gels, USA).

### Phylogenetic analysis of *C. rosea* CDHs

Based on the established protein sequences of fungal CDHs from 14, representing the full extant sequence variation of CDH, the number of sequences was reduced by clustering them for ≥60% sequence identity using USEARCH (35) and representing each cluster by one sequence only. Characterized CDH sequences from literature and six putative CDH sequences from *C. rosea* were added to the selection. After an initial MAFFT G-INS-i alignment (36), all sequences with deletions of > 5 amino acids in otherwise well-conserved regions were deleted, resulting in a selection of 99 sequences. Sequences were re-aligned with MAFFT G-INS-i, and the alignment was trimmed for positions with ≥50% gaps using trimAl (37). The best evolutionary model was inferred to be LG+I + G4+F by modeltest-ng-0.1.6. (https://github.com/ddarriba; accessed on 30.10.2019) under the Akaike Information Criterion. A maximum-likelihood phylogenetic tree was inferred using RAxML-NG v0.9.0 (https://github.com/amkozlov/raxmL-ng; released on 24.05.2019) with a 3% cutoff threshold for the mean relative error-based bootstrap convergence criterion resulting in 520 bootstrap repetitions. The fully annotated tree is available as Fig. S2.

### Modeling

Templates for the six proteins were modeled using the SWISS-MODEL homology-modeling server (38). According to these results, the template with the highest identity for each enzyme was chosen. The Artificial Intelligence-based AlphaFold2 3D-structure prediction system was used through the AlphaFold Colab notebook platform (32). It predicts protein structures based on their sequences using a slightly simplified version of AlphaFold v2.0 with standard settings. The structures of the DH and Cyt domains were predicted individually with exclusion of the linker. The respective templates with the highest coverage (Fig. S3), which was model one in all the cases, were selected for each enzyme, and the structure was analyzed with ChimeraX (39). In ChimeraX, the tool render by attribute was used to visualize the RMSD of the sequence in the AlphaFold structure from *Cr*CDH_A to F compared to *Nc*CDH (PDB: 4qi6) or *Mt*CDH (PDB: 4qi7) (11).

### RNA extraction and transcriptional analysis

Transcriptional analysis of *C. rosea* IK726 under conditions representing all major ecological niches of this fungus, namely, mycotrophy, saprothrophy, and colonization of living plant roots, was performed to categorize the representatives of the expanded gene family encoding for AA3_1 GMC-oxidoreductases according to their transcriptional activity. For fungus-fungus interaction, two time points (sensing the host fungus pre-contact and when interaction is established [40]) were included.

Agar blocks of *C. rosea* IK726 and the host fungus (*Fusarium culmorum* PV8) culture or two agar blocks of *C. rosea* IK726 were inoculated on opposite ends of a cellophane-covered agar plate (to facilitate mycelium harvest) with synthetic Czapek‐Dox minimal medium containing 2% glucose, 0.1% KH_2_PO_4_, 0.3% NaNO_3_, 0.05% KCl, 0.05% MgSO_4_ x 7 H_2_O, 0.002% FeSO_4_ x 7 H_2_O, and 1.5% agar at pH 5.0. Cultivations were performed at 25°C in darkness. Mycelia were harvested from *C. rosea* colonies interacting with *F. culmorum* and with itself a) prior and b) during physical contact between the hyphae, as described in 40. Cultivation in Czapek-Dox agar plates with 2% MC (Sigma, Germany) and ground ST as sole carbon sources was performed essentially as described above, but with the three *C. rosea* colony plugs.

Plant-fungus interaction was investigated using wheat seedlings performed in four biological replicates, and each replicate had five plants for each treatment, following the procedure described previously (33). In brief, surface-sterilized wheat seeds of the cultivar “Stava” were germinated on sterilized moist filter paper within 9-cm-diameter Petri dishes, with 5 seeds allocated to each plate (30). After 3 days of growth, the wheat seedlings were inoculated by dipping their roots in spore *C. rosea* IK726 spore suspension at a concentration of 1 × 10^6^ spores/mL under sterile conditions. Following inoculation, the seedlings were returned to the filter paper in the Petri plates and incubated at a temperature of 20°C. The roots were harvested 4 days post-inoculation and were snap-frozen in liquid nitrogen. Wheat roots inoculated with water were used as a control treatment. Collected mycelia and root samples were ground to a fine powder under liquid nitrogen, and total RNA was extracted with the RNeasy Plant Mini Kit (Qiagen, Germany) followed by further purification using the RNeasy MiniElute Cleanup Kit (Qiagen, Germany). RNA integrity was checked using a Nanodrop Bioanalyzer (Thermo Scientific, Germany). Samples in biological triplicates per each experimental condition were submitted to Mycosynth AG (Basel, Switzerland), where additional quality check of the RNA following poly(A) enrichment or ribosomal RNA depletion was performed. A stranded Illumina cDNA library was created by reverse transcription including the ligation of sequencing adapters with barcodes. Finally, the libraries were pooled and sequenced on the Illumina NovaSeq v2 platform with paired-end reads of 2 × 75 bases length. The fungal samples and the mixtures of the fungal and plant samples were sequenced with 12 and 50 million read pairs per replicate, respectively. Sequenced reads were produced in the standard fastq format that incorporated both sequence information and quality scoring. After quality control, the sequencing reads were mapped to the *C. rosea* IK726 reference genome using the STAR software (41). As input for statistical analysis, the reads that uniquely map to a gene were counted using HTSeq (42). Differentially expressed genes were identified by specialized statistical software DESeq2 (43). Read counts were normalized, and the variance based on the replicates per condition was calculated. Finally, statistical testing was applied to identify differentially expressed genes that were significantly up or downregulated.

While the scope of this study is deliberately limited to CDH genes (see Table S1), we acknowledge the broader context provided by the entire transcriptome which, at this stage, is beyond the scope of the present study.

### Proteomics

As the high number of *cdh* genes in *C. rosea* genome implicated that this mycoparasite is capable of degrading cellulosic substrates, we performed a time-dependent proteomic study on 3% MC. The proteomes were analyzed using tandem MS. The samples were concentrated 30× using Vivaspin vials (Sartorius, Germany). Further, they were reduced (15 mM DTT), carbamidomethylated (55 mM iodoacetamide), and methanol-/chloroform-precipitated, as described by Botelho et al. (44). The pellet was re-dissolved in 0.1 M ammonium bicarbonate buffer and digested overnight with trypsin in a 1:50 enzyme-to-substrate ratio (sequencing grade trypsin, Promega) at 37°C (45, 46). Quantitative shotgun proteomics was performed on an Orbitrap Exactive HF-X instrument equipped with a nanospray source at Max F. Perutz Laboratories (MFPL; Vienna, Austria). A 2-h gradient was applied. The analysis files were converted (using Data Analysis, Bruker) to XML files, which are suitable for performing an MS/MS ion search with GPM (X!-Tandem Algorithm). The data were then assigned to protein sequences of the *C. rosea* protein database, as described by 47. Peptides and proteins were identified at a false discovery rate of 1%.

### Activity assays

Samples for activity assays were prepared by performing buffer exchange and concentrating the supernatants using Vivaflow 200 ultra-centrifuge tube with a 10 kDa molecular weight cut-off. A total volume of 500 µL of each sample was first concentrated at 12,000 g at room temperature to reach a sample volume of 50 µL. Citrate-phosphate buffer (pH 5.5) was added to the concentrated sample to reach a final volume of 500 µL, followed by a second concentration step, reducing it to 50 µL. This process was repeated three times, ultimately resulting in a final sample volume of 500 µL.

The activity assays were performed essentially as described by (48) using DCIP as an electron acceptor. Briefly, the reaction mixture contained 5 mM DCIP in 20 mM citrate phosphate buffer pH 5.5. The substrates were tested using 50 mM concentration, except for glucose and mannose that were tested also at 500 mM. The absorbance of the reaction mixture was measured at 520 nm at 2 min intervals after adding substrate for 24 h in POLARStar Omega (BMG Labtech, Germany) maintaining a constant temperature of 30°C. Initially, the DCIP assays were performed in 96-well FF Biolog plates (Biolog, US), coated with 95 different substrates (monosaccharides, polysaccharides, oligosaccharides, glucosides, sugar acids, hexosamines, polyols, nitrogen-containing compounds, etc.), and water (see Table S2). These substrates were tested with *Cr*CDH_A to F in their supernatant and a negative control consisting of nontransformed *K. phaffii* X33 supernatant. Substrates that showed promising activity in the initial activity screening were assayed in triplicates again including *Nc*CDH2 as a positive control, using a defined concentration of 5 µmol (11). All the data were analyzed using MARS version 3.3 R 5 software (BMG Labtech, Germany) and GraphPad Prism version 10.1.2 software (Dotmatics).

### Mass Spectrometry

The activity assays of *Cr*CDH_A and B and *Nc*CDH, using the sugars glucose, mannose, maltose, cellobiose, lactose, and maltotriose as substrates, showed a positive activity and were analyzed using MS after filtering through 10-kDA cut off Vivaspin 500 spin columns (Sartorius, Germany). High-resolution electrospray ionization orbitrap mass spectrometry (ESI-orbitrap-MS) was used to identify the oxidized oligosaccharides as sodium adducts [M-H Na]. The products were separated using a Vanquish Horizon UHPLC system (Thermo Fisher Scientific) equipped with a porous graphite-based Hypercarb 4.6 ⨯ 100 mm column with a 3 μm particle size (Thermo Scientific, Waltham, MA, USA), and the column compartment was maintained at 40°C. The mobile phase consisted of two solvents: eluent A (water with 0.2% ferulic acid) and eluent B (acetonitrile with 0.2% ferulic acid). Separation was achieved using a gradient from 0% to 20% of eluent B at a flow rate of 0.5 mL/min, as follows: The initial phase of separation, spanning 0 to 6 min, was conducted with an acetonitrile concentration ranging from 0% to 20%. Thereafter, a constant level of 20% acetonitrile was held for 5 min. Afterward, a gradient of eluent B from 20% to 0% over 1 min was used, followed by a cleaning and column equilibration phase of 100% A for 2 min. The Q Exactive HF mass spectrometer was operated in full scan positive ion mode (scan range 100–1,500 *m/z*) with a spray voltage of 3.5 kV, a capillary temperature of 350°C, and sheath and auxiliary gas pressures of 45 and 15 arbitrary units, respectively. MS spectra were acquired at a resolution of 120,000 with an auto-gain control target of 1 × 10^6^. The spectra were analyzed using Freestyle 1.7 available in the program Thermo Scientific Xcalibur (Thermo Scientific, Bremen, Germany).

## Data Availability

The accession numbers of all *C. rosea cdh* genes used in this study are available in the Materials and Methods and in the Table S1. The genome of *C. rosea* IK726 is available in the JGI MycoCosm fungal genomic resource (https://mycocosm.jgi.doe.gov/Cloros1/Cloros1.home.html). All data supporting the findings of this study are available within the published article and its supplementary materials.

## SUPPLEMENTAL MATERIAL

Supplemental figures spectrum.02577-25-s0001.pdf Fig. S1 to S19.

Table S1 spectrum.02577-25-s0002.xlsx Normalized transcript counts and DESeq2 statistics for *cdh* genes across multiple pairwise comparisons.

Table S2 spectrum.02577-25-s0003.xlsx DCIP-based enzymatic activity prescreening for *Cr*CDH supernatant variants, negative control, and blank using FF Biolog plates precoated with 95 substrates and water.

ASM does not own the copyrights to Supplemental Material that may be linked to, or accessed through, an article. The authors have granted ASM a non-exclusive, world-wide license to publish the Supplemental Material files. Please contact the corresponding author directly for reuse.
