# Supplementary material for: Structural insights into cellobiose dehydrogenases of a non-lignocellulolytic fungus and their transcriptional dynamics across different nutritional modes
Source: Microbiol Spectr. 2025 Dec 31;14(2):e02577-25. doi: 10.1128/spectrum.02577-25 (PMC12889107; doi:10.1128/spectrum.02577-25)
Supplement: Supplemental figures — Fig. S1 to S19. [file spectrum.02577-25-s0001.pdf]

Structural insights into cellobiose dehydrogenases of a non-lignocellulolytic fungus and their transcriptional dynamics across different nutritional modes

Naike Kruhler[1], Sarbagya Ratna Shakya[1], Mukesh Dubey[2], Leander Sützl[3], Clemens Peterbauer[3], Magnus Karlsson[2], Mats Sandgren[1], Lea Atanasova[1,3,4]*

[1] Department of Molecular Sciences, Swedish University of Agricultural Sciences, Uppsala, Sweden.

[2] Department of Forest Mycology and Plant Pathology, Swedish University of Agricultural Sciences, Uppsala, Sweden.

[3] Department of Biotechnology and Food Science, BOKU University, Vienna, Austria.

[4] Department of Agricultural Sciences, BOKU University, Tulln, Austria.

*Corresponding author: Lea Atanasova; lea.atanasova@boku.ac.at

Supplementary Figures

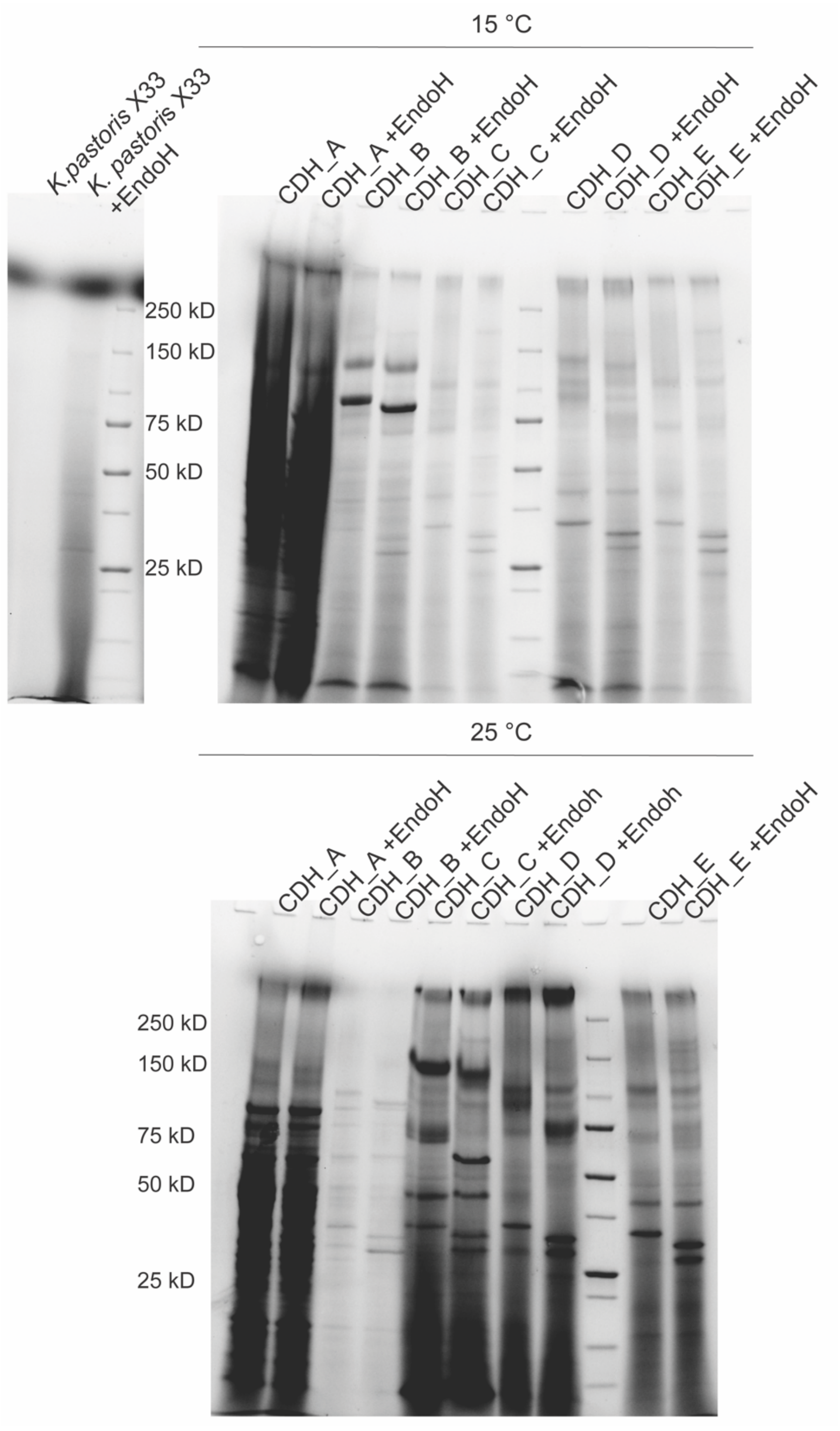

Supplementary Figure 1: Cultivation of *Cr*CDH_A to E at 15 °C and 25 °C at pH 5.5. SDS-PAGE analysis of recombinant *Cr*CDH_A to E expression in *K. phaffii* under different cultivation conditions. The yeast cells were first selected on YPG plates containing 500 mg/mL zeocin and then cultivated in liquid YPG medium following methanol induction according to Invitrogen's Pichia Fermentation Guidelines. Expression cultures were incubated for six days at either 15°C or 25°C with methanol induction. After harvesting, the culture supernatants were concentrated using Vivaflow 200 (10 kDa cut-off). The gel displays protein bands corresponding to *Cr*CDH_A to E, with and without Endoglycosidase H (Endo H) treatment, to assess potential glycosylation. Differences in band patterns across conditions provide insights into expression efficiency and concentration estimation of the recombinant enzymes.

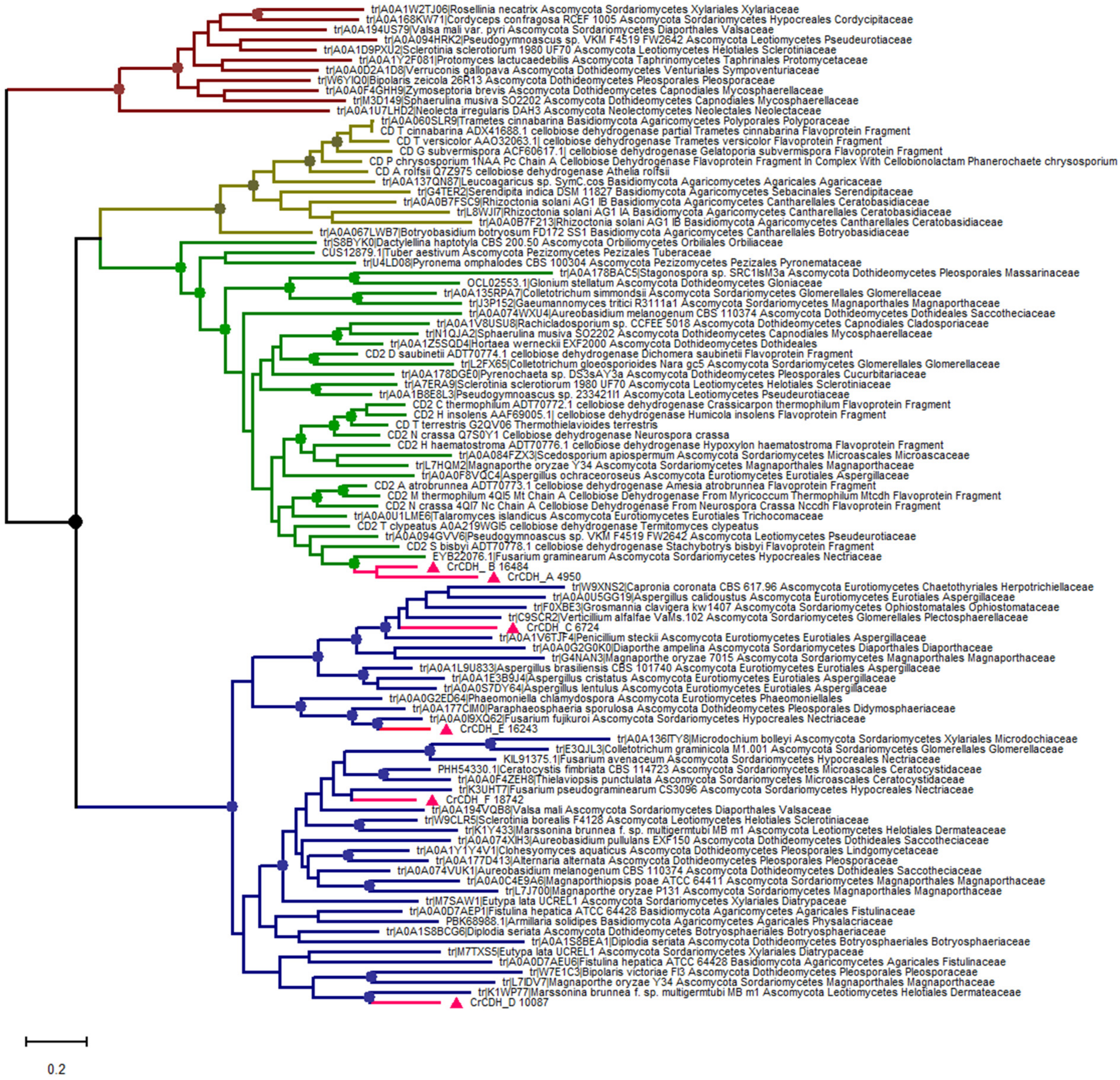

Supplementary Figure 2: Detailed maximum likelihood (ML) phylogenetic tree based on the established sequence selection of fungal CDHs. A ML tree was inferred using RAxMLNG v0.9.0 with a 3 % cutoff threshold for the MRE-based bootstrap convergence criterion resulting in 520 bootstrap repetitions. The nodes supported by bootstrap values larger than 95% are marked with dots.

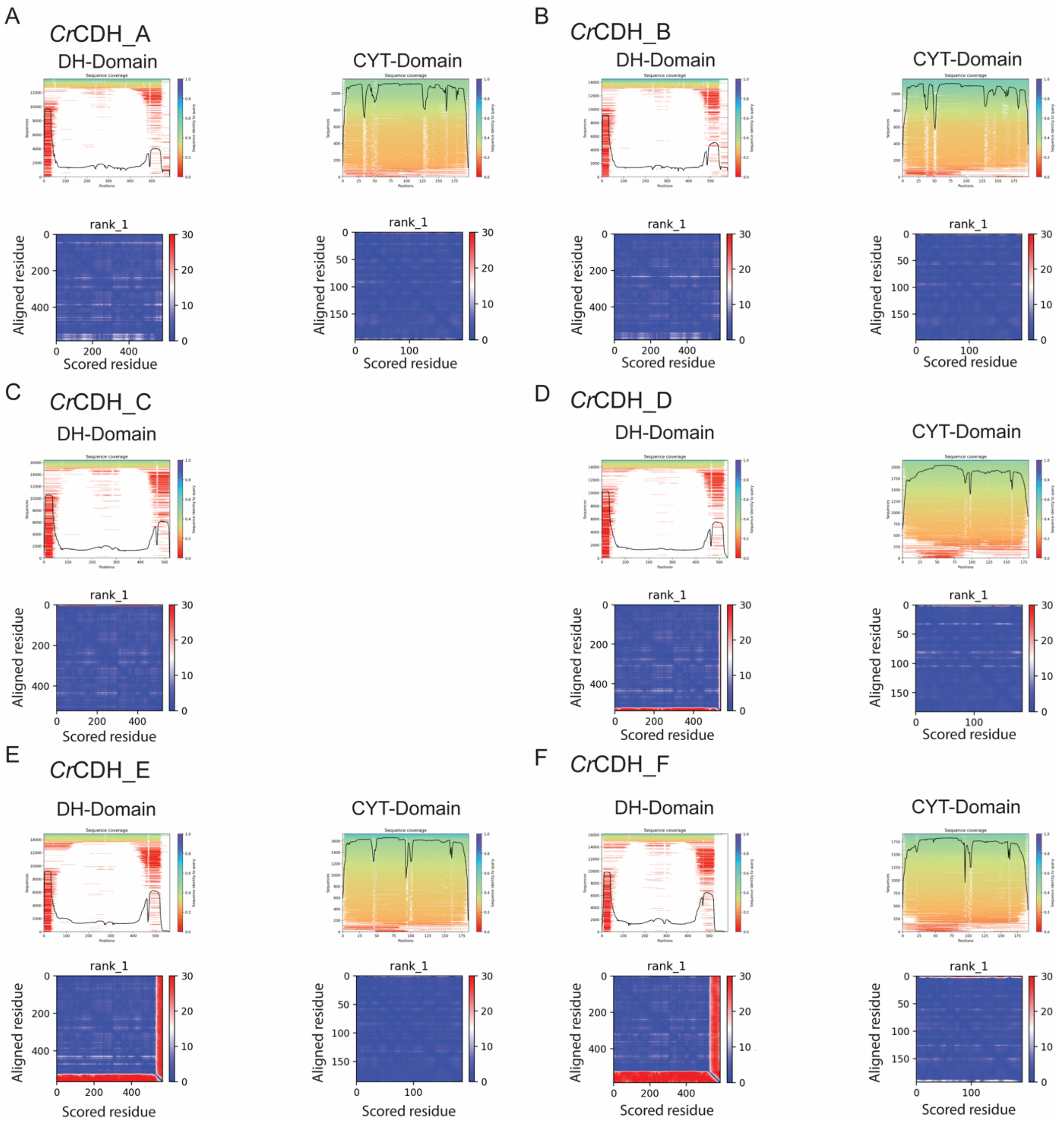

Supplementary Figure 3: The Alphafold2 prediction plots of *Cr*CDH_A to F are displayed. The plot on the top shows the multiple sequence alignment of *Cr*CDH_A to F with its template, and the plot at the bottom shows the predicted alignment error for each residue in the predicted structures, with a comparison to a residue in the true structure. A) B) *Nc*CDH (PDB: 4qi6) was employed as a template for *Cr*CDH_A and B. C) *Cr*CDH_C Dehydrogenase (DH) domain was predicted with *Mt*CDH (PDB: 4qi7). D) The DH and (cytochrome) CYT domains of *Cr*CDH_D were predicted with the template *Mt*CDH (PDB: 4qi7). E) The DH and CYT domains of *Cr*CDH_E were predicted with the template *Nc*CDH (PDB: 4qi6).

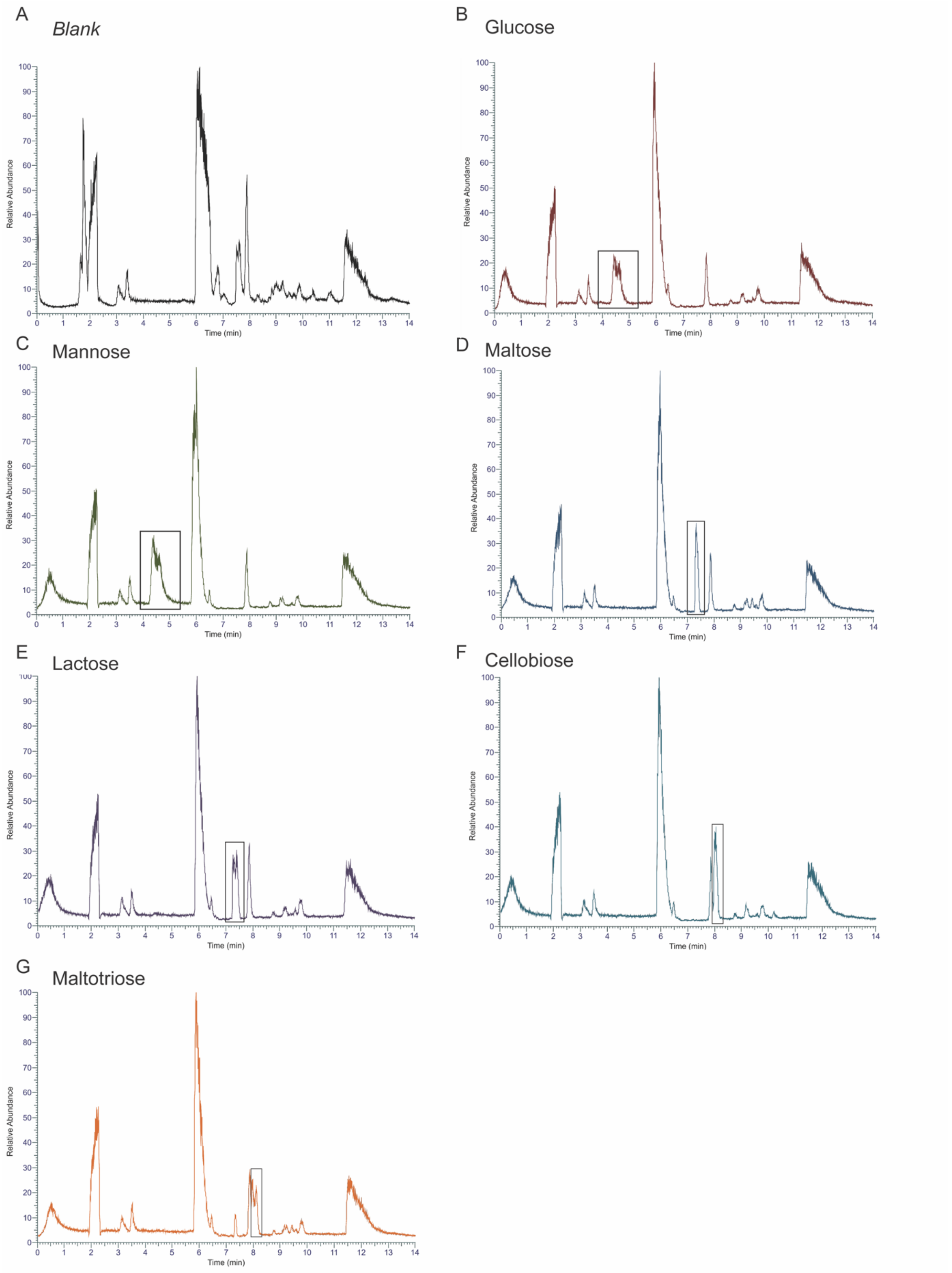

Supplementary Figure 4: Comparison of LC-chromatograms of the sugars utilized in the activity assays. The samples are as follows: A) blank, B) glucose (4.5 min), C) mannose (4.5 min), D) maltose, (7.4 min), E) lactose (7.4 min), F) cellobiose (8.1 min), G) maltotriose (8.1 min). The peaks corresponding to the sugars are indicated in each chromatogram with a black box.

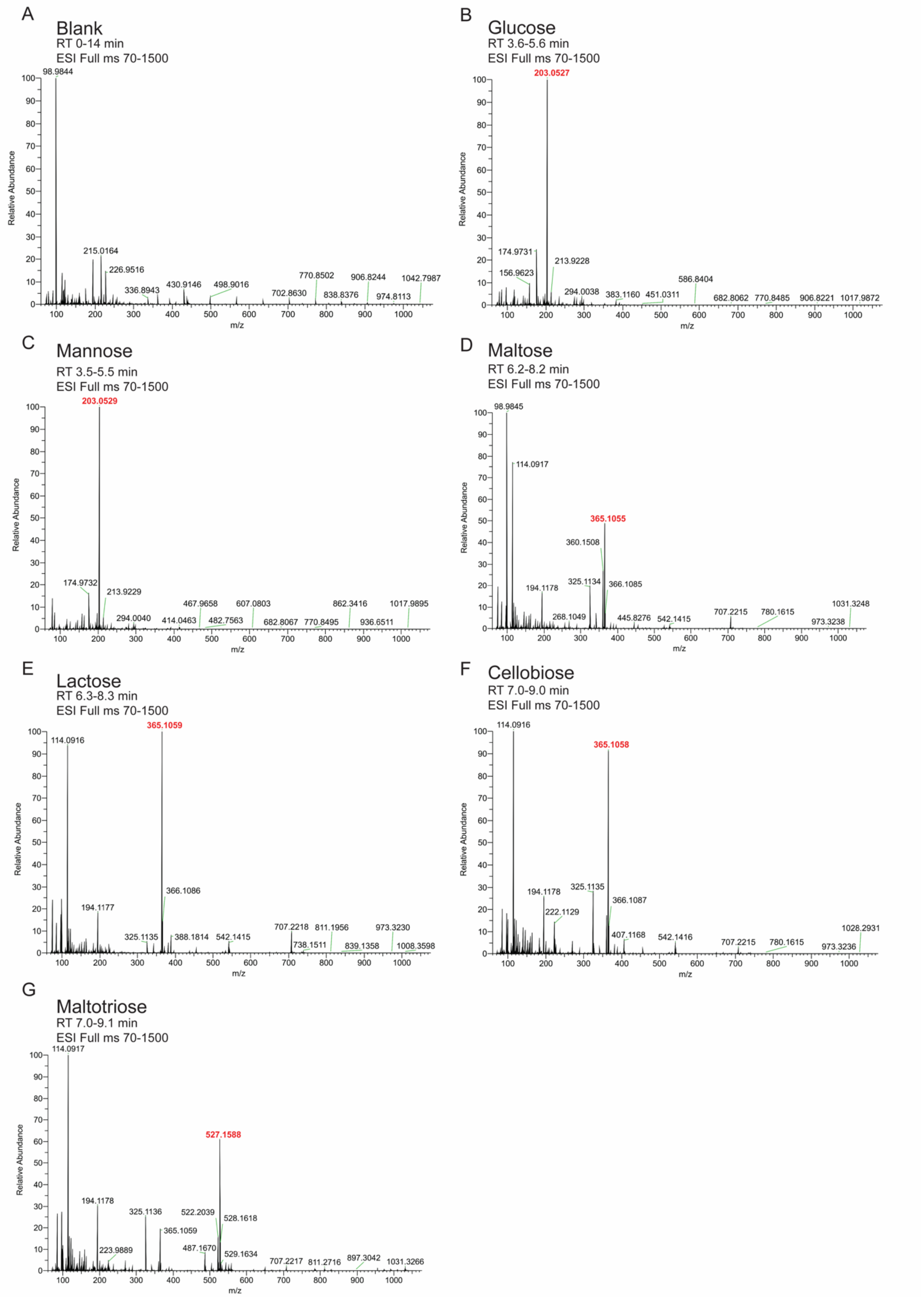

Supplementary Figure 5: MS spectra collected in positive ion mode showing the ionization of the sugars tested for activity. The spectra show is averaged over a retention time (RT) approx. +1 to -1 minute before and after the respective substrate eluted in the chromatogram. The sugars are detected as sodium adducts with glucose (4.5 min, m/z 203), mannose (4.5 min, m/z 203), maltose (7.4 min, m/z 365), lactose (7.4 min, m/z 365), cellobiose (8.1 min, m/z 365), and maltotriose (8.1 min, m/z 527)

eluted from the liquid chromatography column. For the control no time was selected and the full scan is displayed.

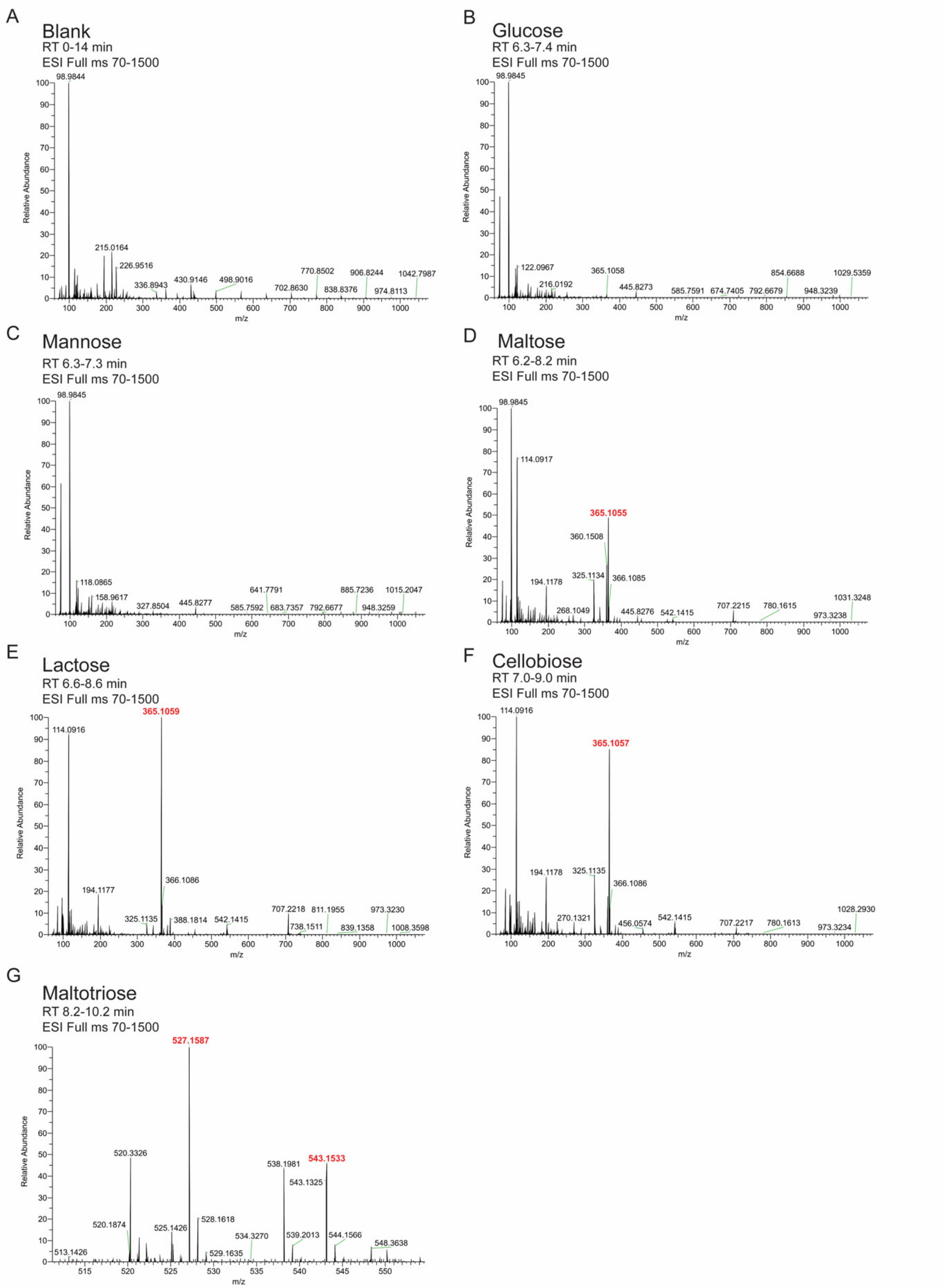

Supplementary Figure 6: MS spectra collected in positive ion mode showing the ionization of the sugars tested for activity. The spectra show is averaged over a retention time (RT) approx. +1 to -1 minute before and after the respective oxidized sugar eluted in the chromatogram. No oxidized sugar was detected except for maltotriose (9.2 min, m/z 543). For the control no time was selected and the full scan is displayed.

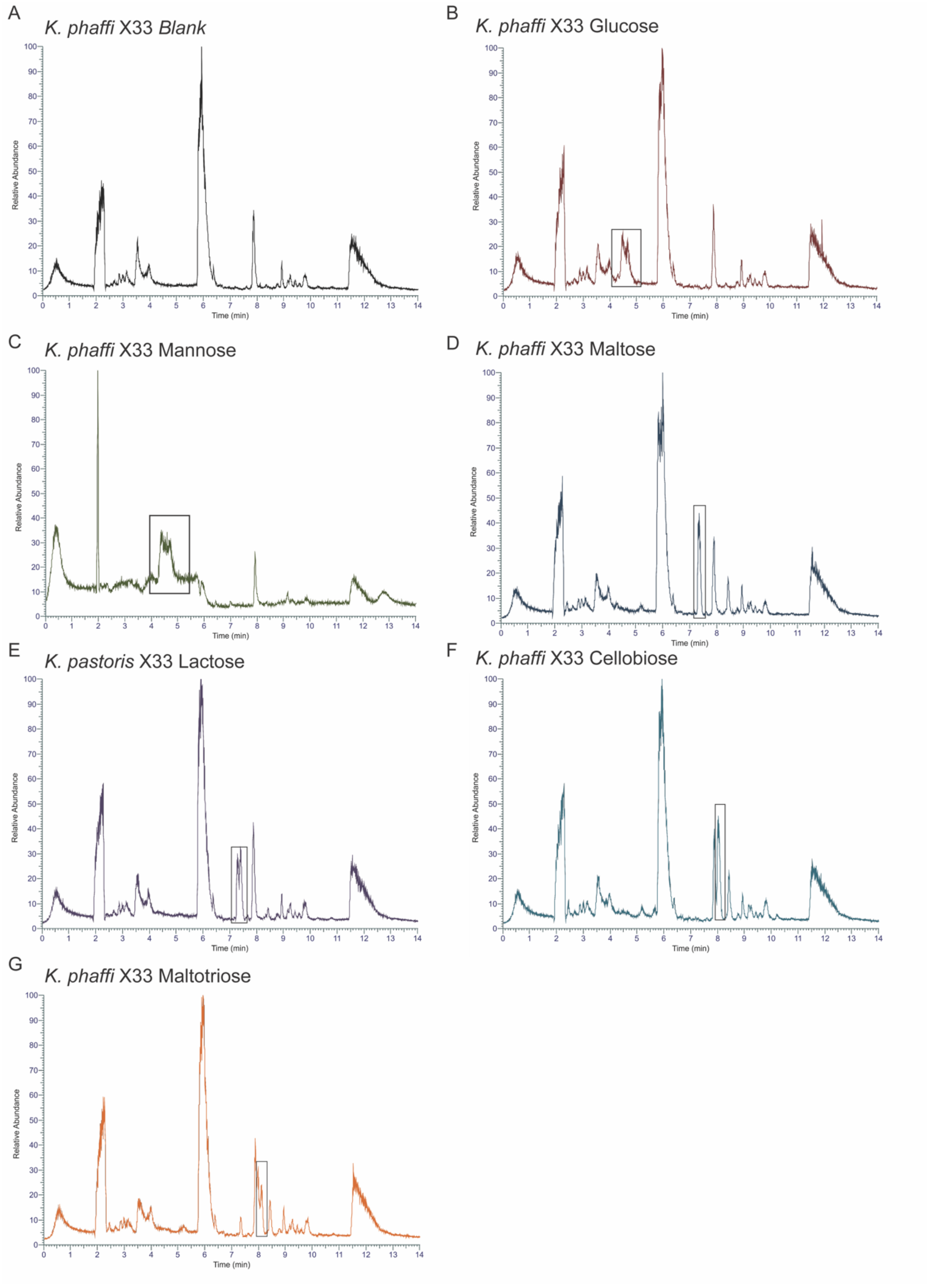

Supplementary Figure 7: Comparison of LC-chromatograms of the *K.phaffi* X33 supernatant activity assays, as a control with the tested sugars. The samples are as follows: A) blank, B) glucose (4.5 min), C) mannose (4.5 min), D) maltose, (7.4 min), E) lactose (7.4 min), F) cellobiose (8.1 min), G) maltotriose (8.1 min). The peaks corresponding to the sugars are indicated in each chromatogram with a black box.

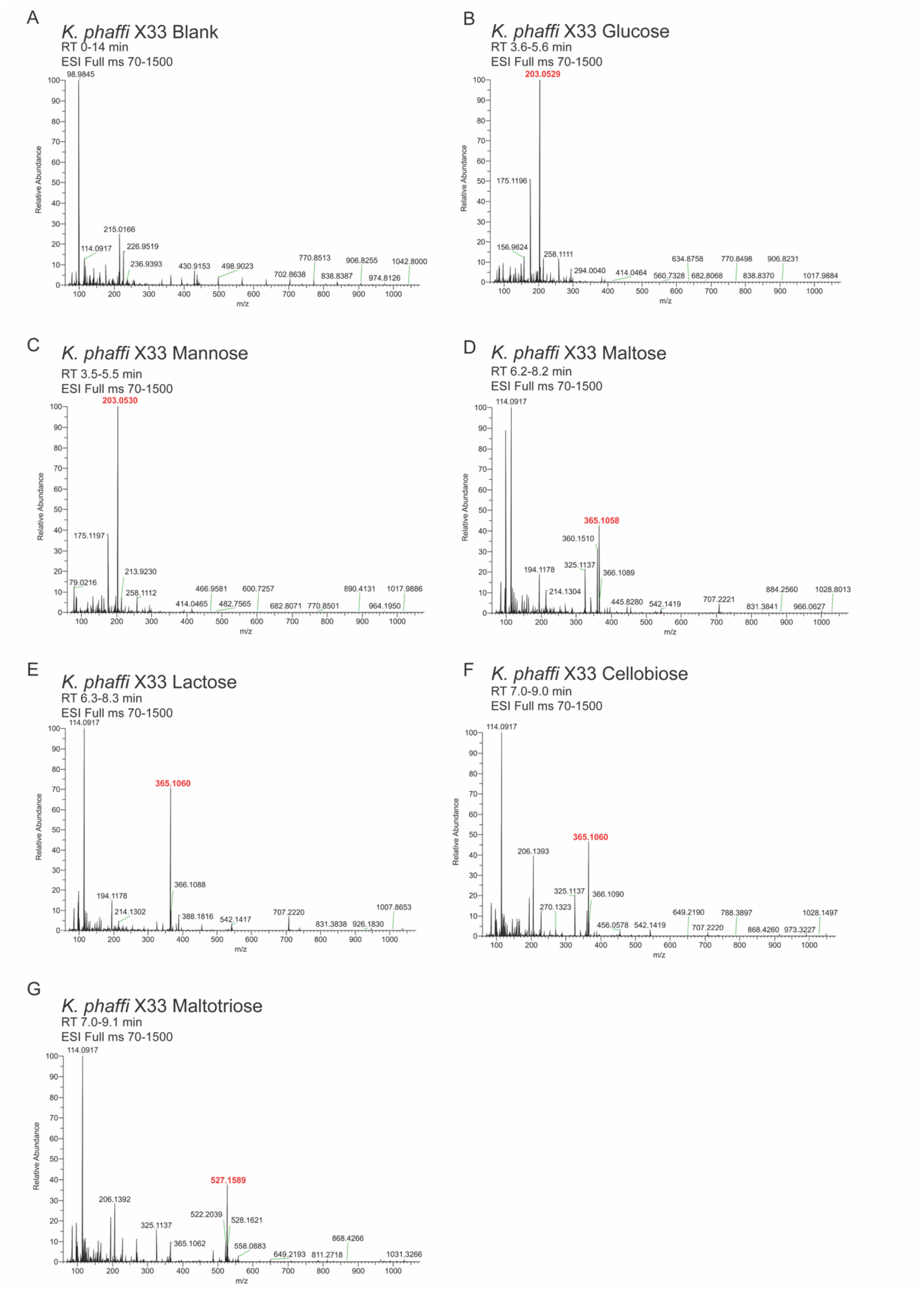

Supplementary Figure 8: MS spectra collected in positive ion mode showing the ionization of the activity assay with *K. phaffii* X33 supernatant and the tested sugars. The spectra show is averaged over a retention time (RT) approx. +1 to -1 minute before and after the respective substrate eluted in the chromatogram. The sugars are detected as sodium adducts with glucose (4.5 min, m/z 203), mannose (4.5 min, m/z 203), maltose (7.4 min, m/z 365), lactose (7.4 min, m/z 365), cellobiose (8.1 min, m/z

365), and maltotriose (8.1 min, m/z 527) eluted from the liquid chromatography column. For the control no time was selected and the full scan is displayed.

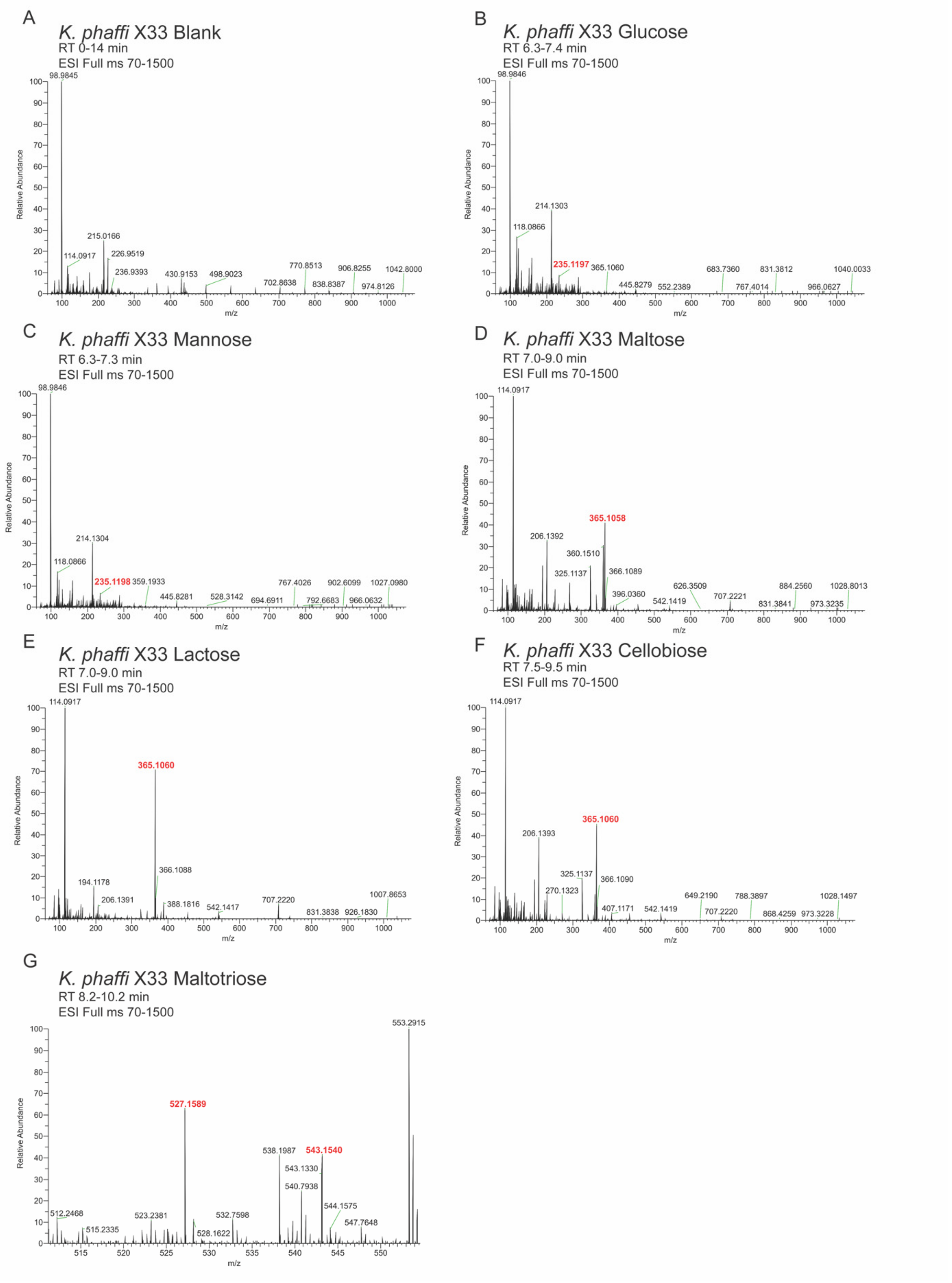

Supplementary Figure 9: MS spectra collected in positive ion mode showing the ionization of the activity assay with *K. phaffii* X33 supernatant and the tested sugars. The spectra show is averaged over a retention time (RT) approx. +1 to -1 minute before and after the respective oxidized sugar eluted in the chromatogram. Oxidized sugar was detected for B) glucose (6.8 min, m/z 235), C) mannose (7.1,

m/z 235) and G) maltotriose (9.2 min, m/z 543). For the control no time was selected and the full scan is displayed.

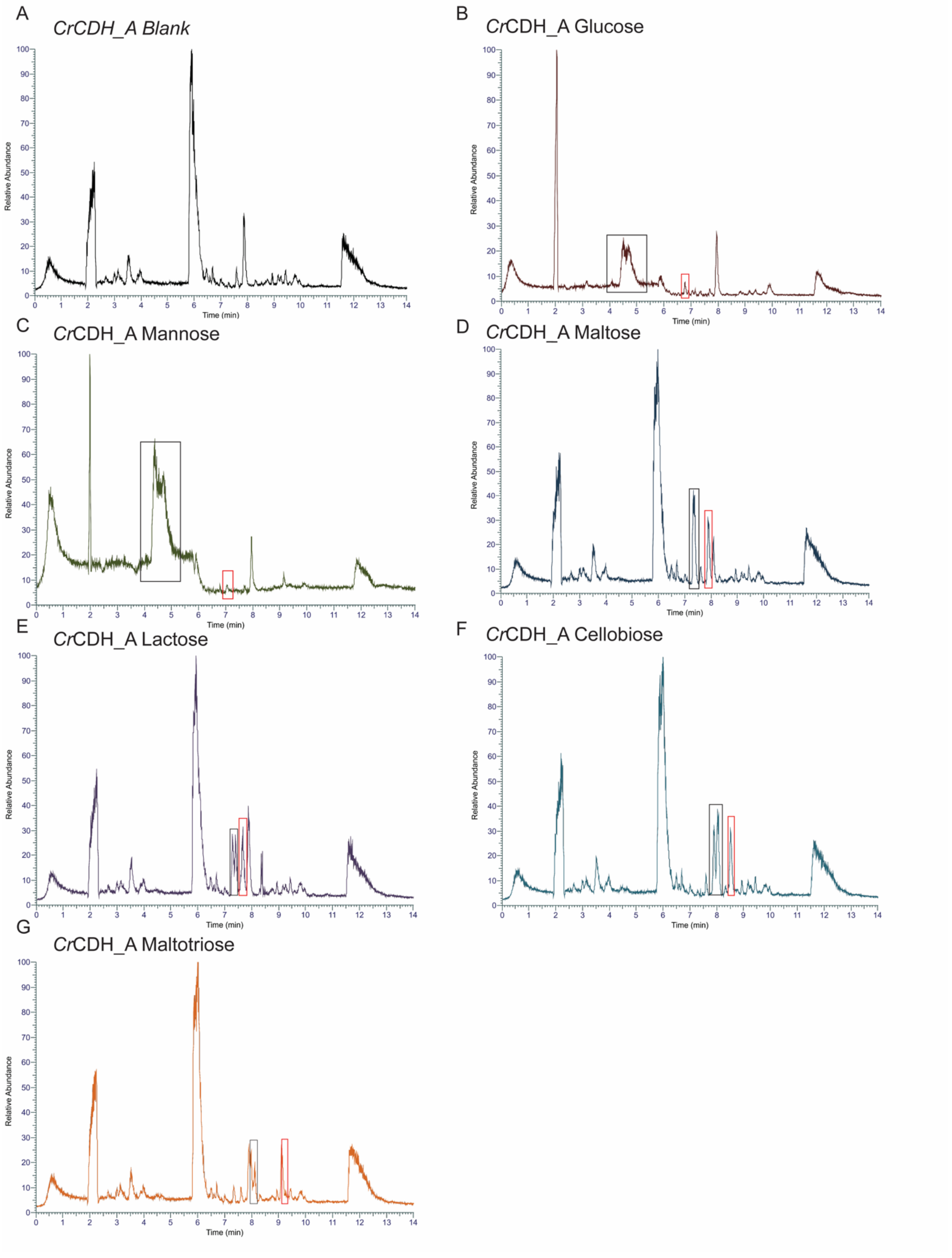

Supplementary Figure 10: Comparison of LC-chromatograms of the sugars utilized in the activity assays incubated with *Cr*CDH_A supernatant. The substrates are as follows: A) blank, B) glucose (4.5 min), C) mannose (4.5 min), D) maltose, (7.4 min), E) lactose (7.4 min), F) cellobiose (8.1 min), G) maltotriose (8.1 min). The substrate peaks corresponding to the sugars are indicated in each chromatogram with a black box. The oxidized products are as follows: A) blank, B) glucose (6.8 min),

C) mannose (7.1 min), D) maltose (7.9 min), E) lactose (7.7 min), F) cellobiose (8.5 min), G) maltotriose (9.2 min). The product peaks are indicated with a red box.

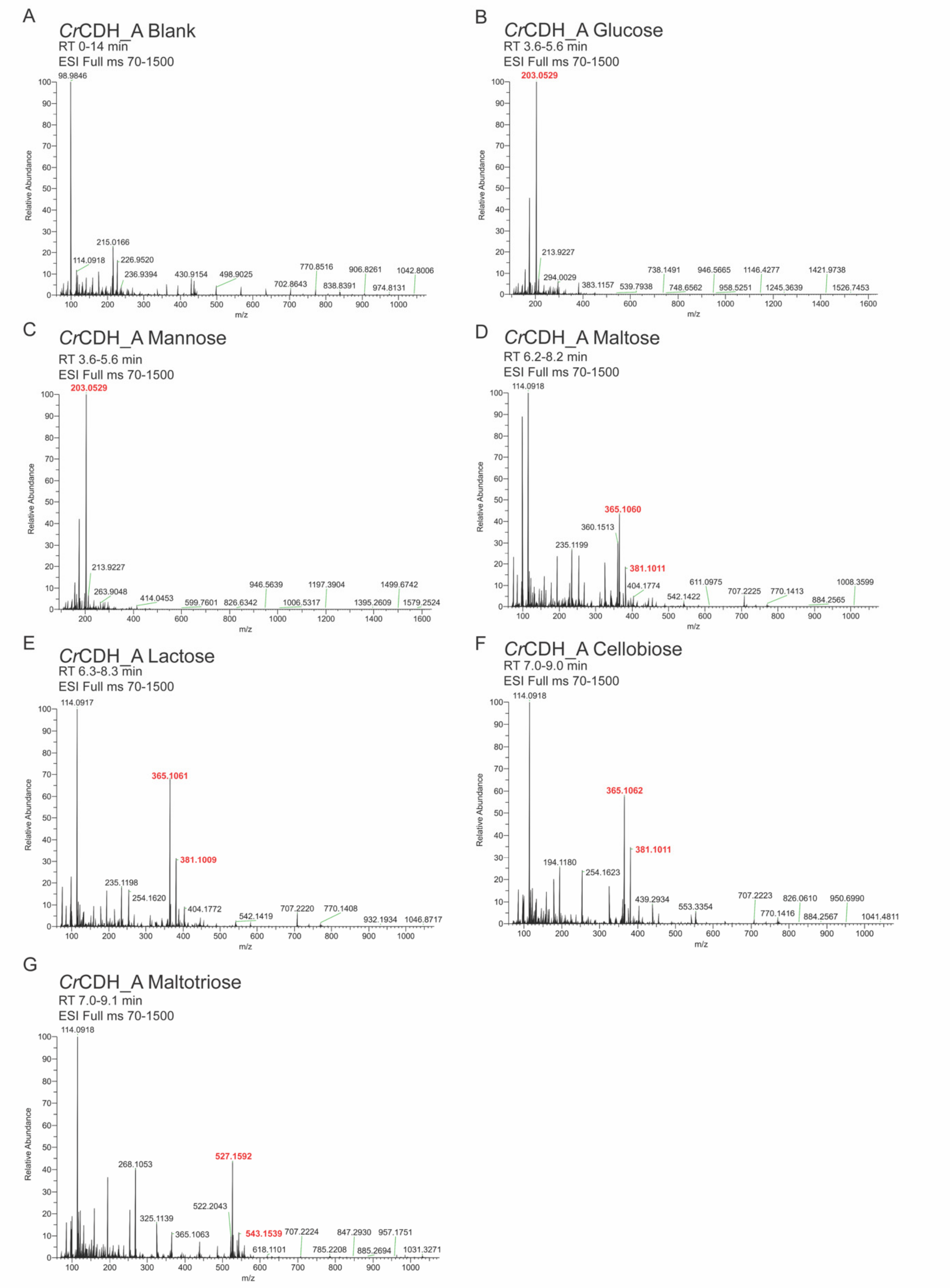

Supplementary Figure 11: MS spectra collected in positive ion mode showing the ionization of the activity assay with *Cr*CDH_A supernatant and the tested sugars. The spectra show is averaged over a retention time (RT) approx. +1 to -1 minute before and after the respective substrate eluted in the chromatogram. The sugars are detected as sodium adducts with glucose (4.5 min, m/z 203), mannose

(4.5 min, m/z 203), maltose (7.4 min, m/z 365), lactose (7.4 min, m/z 365), cellobiose (8.1 min, m/z 365), and maltotriose (8.1 min, m/z 527) eluted from the liquid chromatography column. For the control no time was selected and the full scan is displayed.

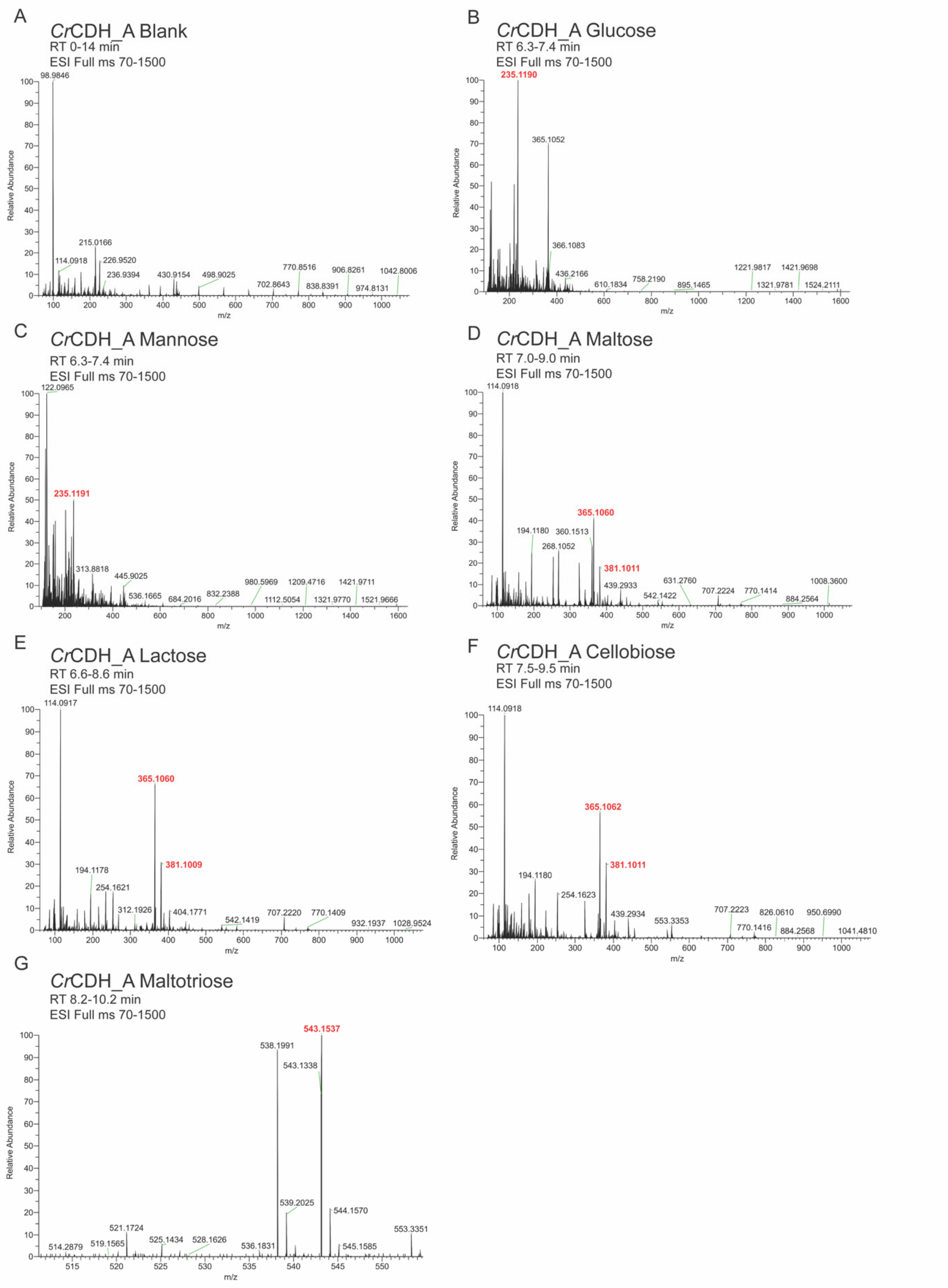

Supplementary Figure 12: MS spectra collected in positive ion mode showing the ionization of the activity assay with *Cr*CDH_A supernatant and the tested sugars. The spectra show is averaged over a retention time (RT) approx. +1 to -1 minute before and after the respective oxidized sugar eluted in the

chromatogram. Oxidized sugar were detected for B) glucose (6.8 min, m/z 235), C) mannose (7.1, m/z 235), D) maltose, (7.9 min,381), E) lactose (7.7 min, 381), F) cellobiose (8.5 min, 381), and G) maltotriose (9.2 min, m/z 543). For the control no time was selected and the full scan is displayed.

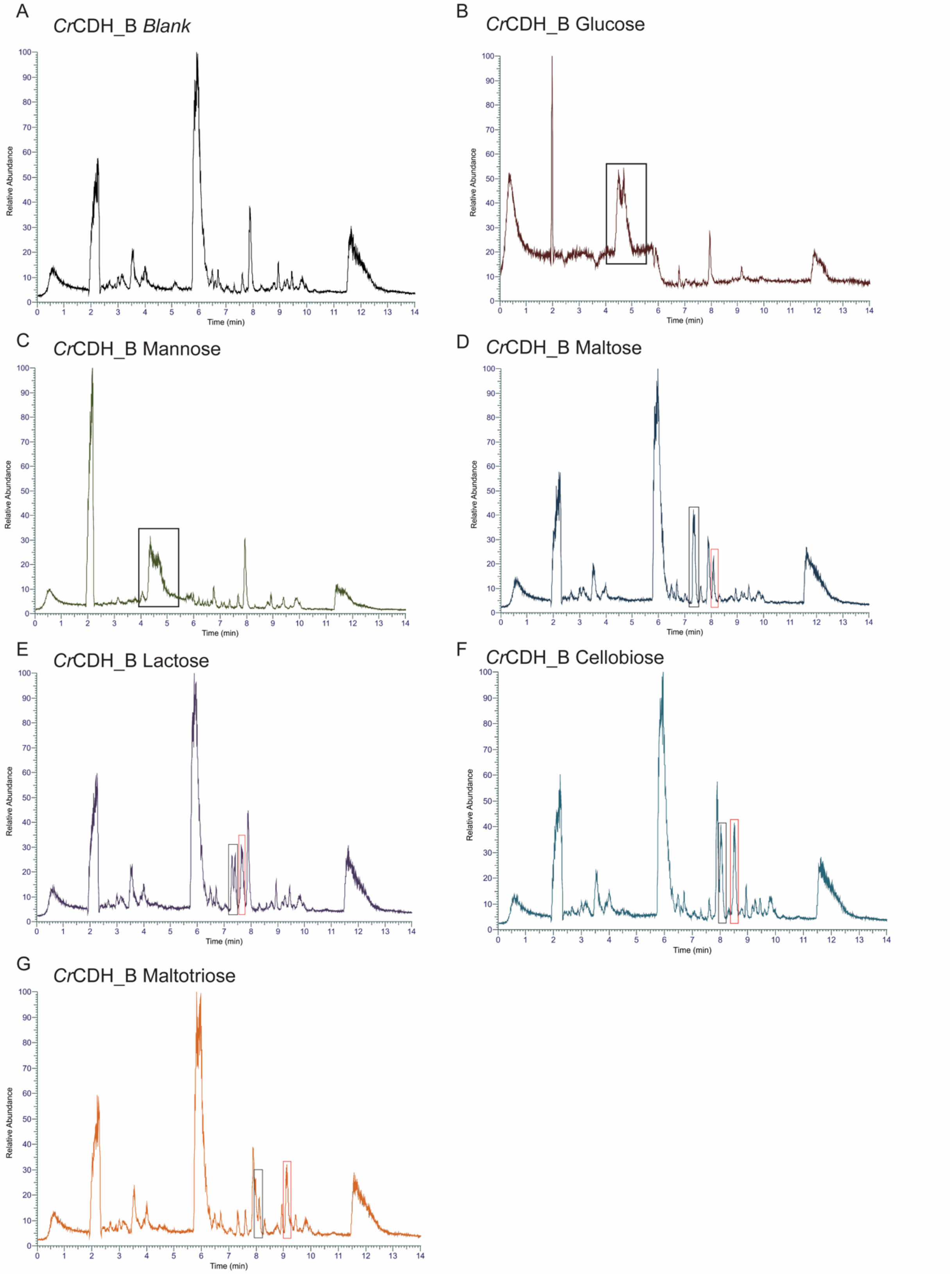

Supplementary Figure 13: Comparison of LC-chromatograms of the sugars utilized in the activity assays incubated with *Cr*CDH_B supernatant. The substrates are as follows: A) blank, B) glucose (4.5 min), C) mannose (4.5 min), D) maltose, (7.4 min), E) lactose (7.4 min), F) cellobiose (8.1 min), G)

maltotriose (8.1 min). The substrate peaks corresponding to the sugars are indicated in each chromatogram with a black box. The oxidized products are as follows: A) blank, B) glucose (6.8 min), C) mannose (7.1 min), D) maltose (7.9 min), E) lactose (7.7 min), F) cellobiose (8.5 min), G) maltotriose (9.2 min). The product peaks are indicated with a red box.

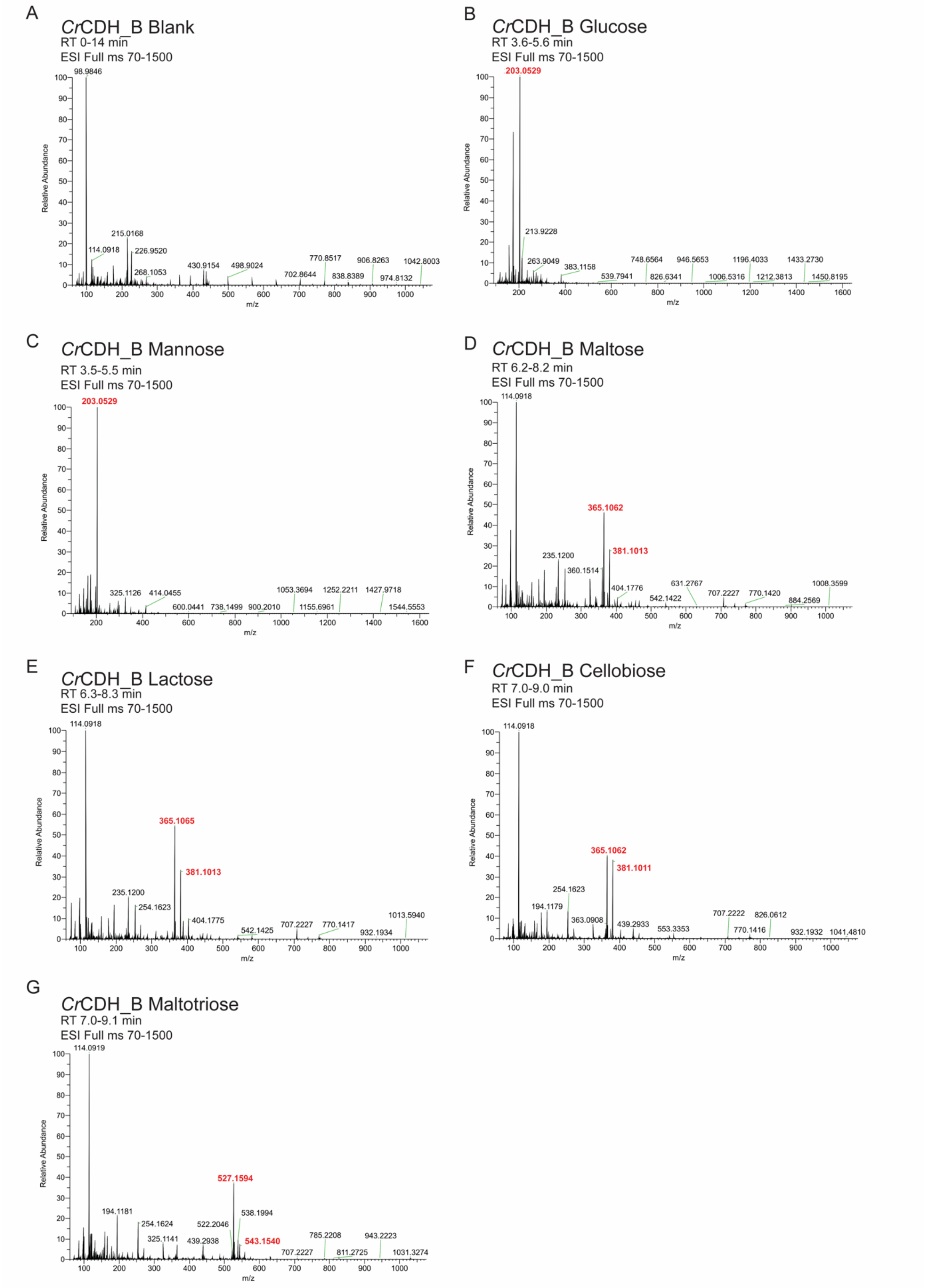

Supplementary Figure 14: MS spectra collected in positive ion mode showing the ionization of the

activity assay with *Cr*CDH_B supernatant and the tested sugars. The spectra show is averaged over a retention time (RT) approx. +1 to -1 minute before and after the respective substrate eluted in the chromatogram. The sugars are detected as sodium adducts with glucose (4.5 min, m/z 203), mannose (4.5 min, m/z 203), maltose (7.4 min, m/z 365), lactose (7.4 min, m/z 365), cellobiose (8.1 min, m/z 365), and maltotriose (8.1 min, m/z 527) eluted from the liquid chromatography column. For the control no time was selected and the full scan is displayed.

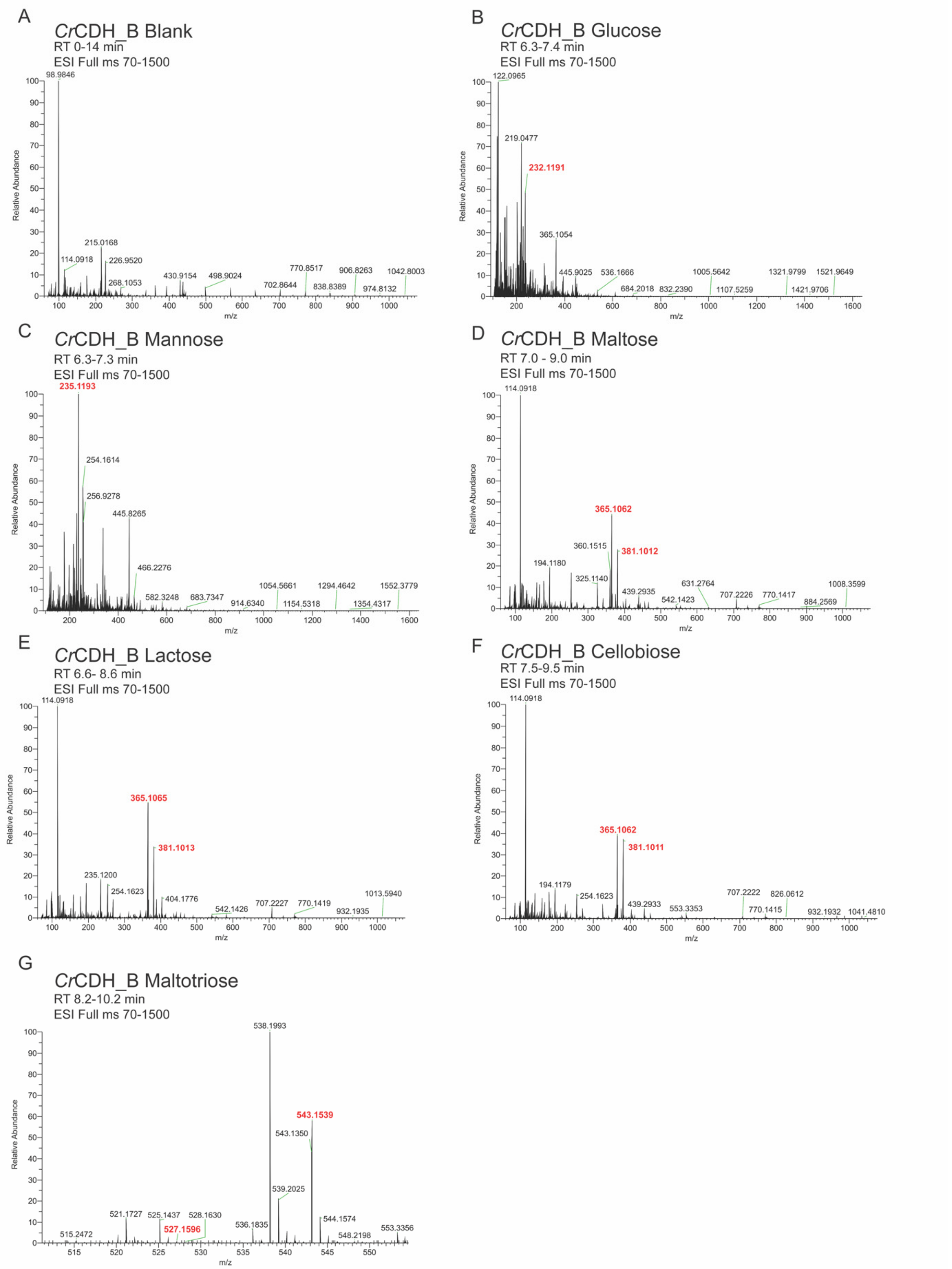

Supplementary Figure 15: MS spectra collected in positive ion mode showing the ionization of the

activity assay with *Cr*CDH_B supernatant and the tested sugars. The spectra show is averaged over a retention time (RT) approx. +1 to -1 minute before and after the respective oxidized sugar eluted in the chromatogram. Oxidized sugar were detected for B) glucose (6.8 min, m/z 235), C) mannose (7.1, m/z 235), D) maltose, (7.9 min,381), E) lactose (7.7 min, 381), F) cellobiose (8.5 min, 381), and G) maltotriose (9.2 min, m/z 543). For the control no time was selected and the full scan is displayed.

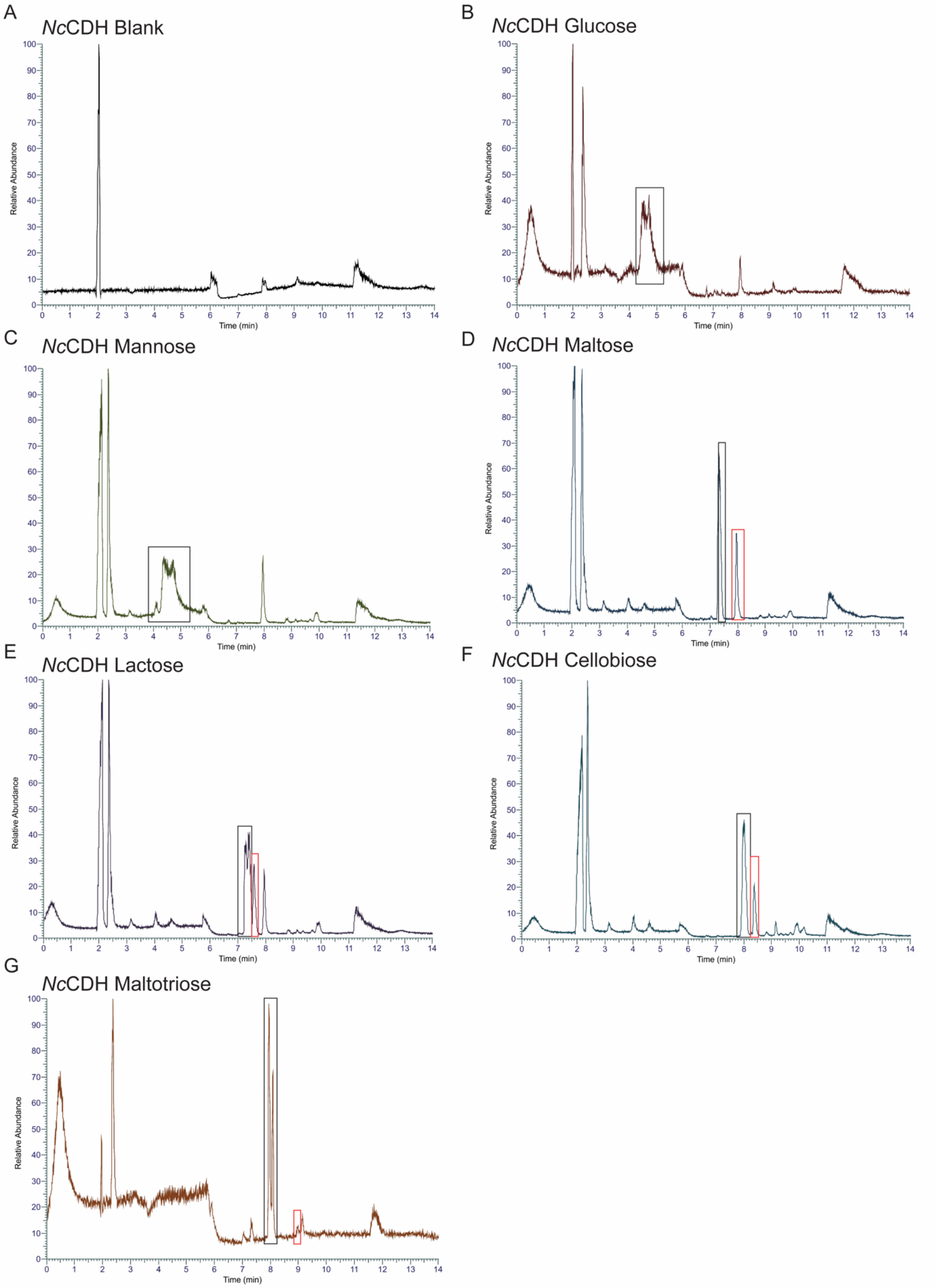

Supplementary Figure 16: Comparison of LC-chromatograms of the sugars utilized in the activity

assays incubated with *Nc*CDH as a positive controll. The substrates are as follows: A) blank, B) glucose (4.5 min), C) mannose (4.5 min), D) maltose, (7.4 min), E) lactose (7.4 min), F) cellobiose (8.1 min), G) maltotriose (8.1 min). The substrate peaks corresponding to the sugars are indicated in each chromatogram with a black box. The oxidized products are as follows: D) maltose, (7.9 min), E) lactose (7.7 min), F) cellobiose (8.5 min), G) maltotriose (9.2 min). The product peaks are indicated with a red box.

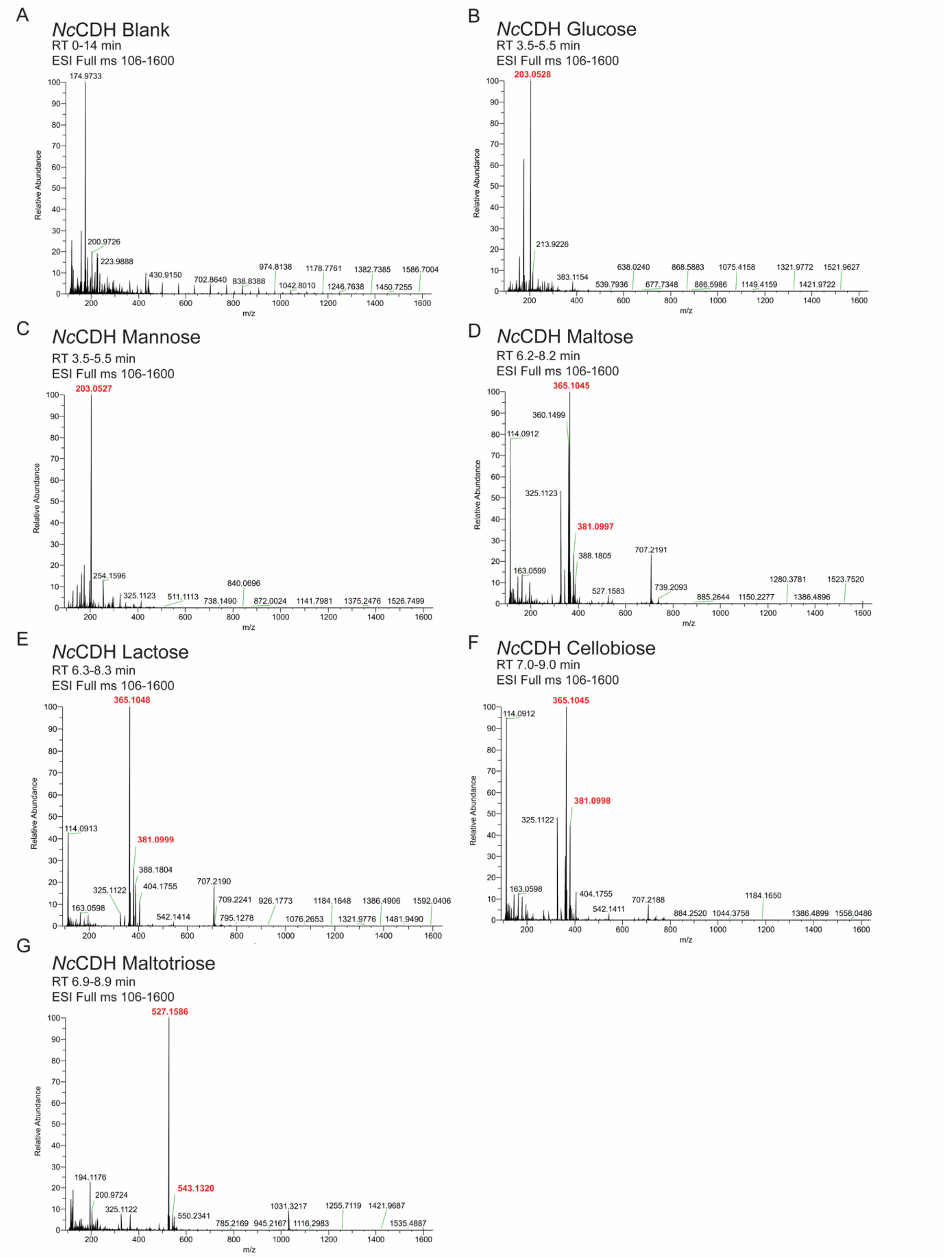

Supplementary Figure 17: MS spectra collected in positive ion mode showing the ionization of the

activity assay with *Nc*CDH purified protein and the tested sugars. The spectra show is averaged over a retention time (RT) approx. +1 to -1 minute before and after the respective substrate eluted in the chromatogram. The sugars are detected as sodium adducts with glucose (4.5 min, m/z 203), mannose (4.5 min, m/z 203), maltose (7.4 min, m/z 365), lactose (7.4 min, m/z 365), cellobiose (8.1 min, m/z 365), and maltotriose (8.1 min, m/z 527) eluted from the liquid chromatography column. For the control no time was selected and the full scan is displayed.

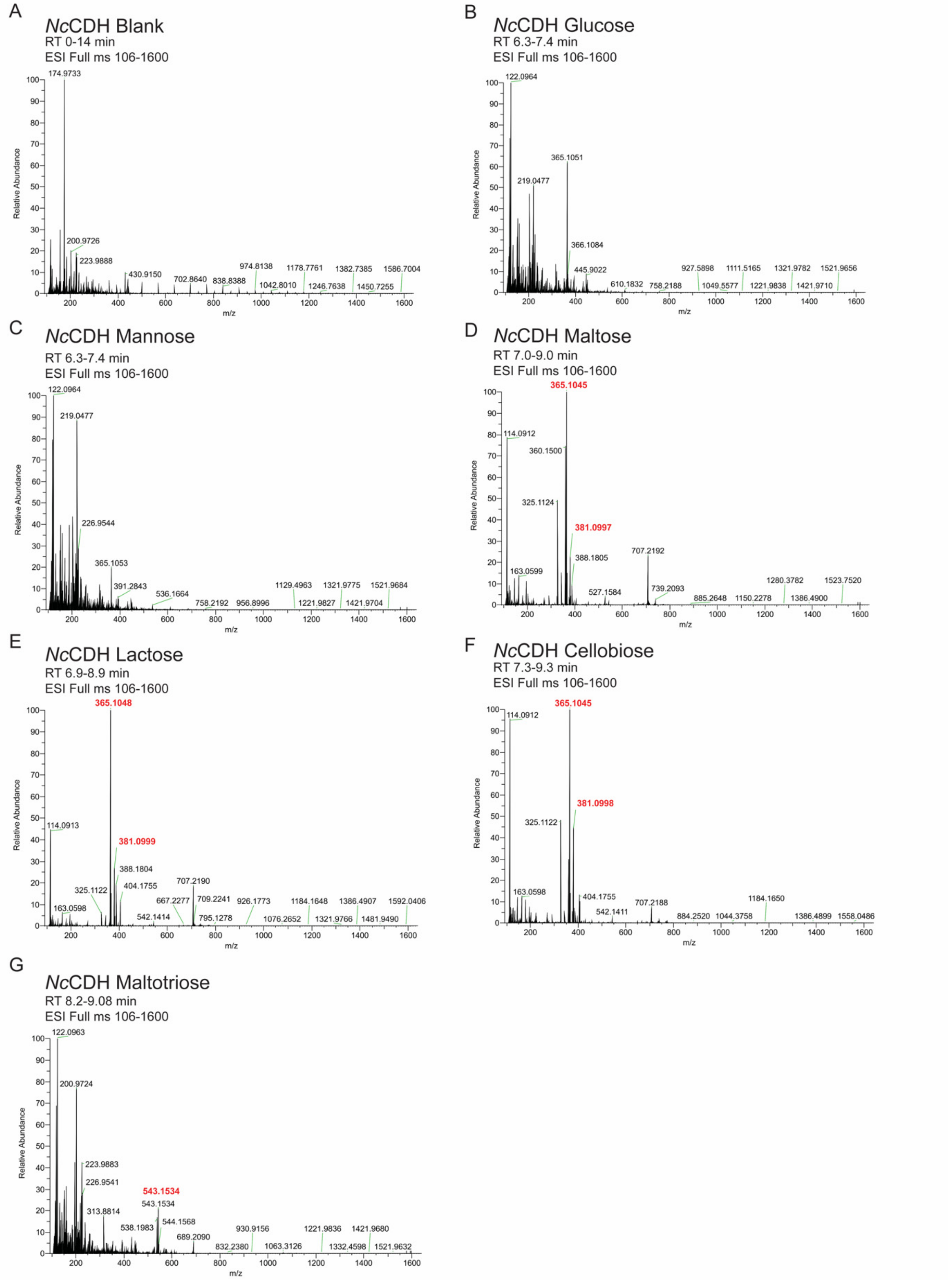

Supplementary Figure 18: MS spectra collected in positive ion mode showing the ionization of the

activity assay with purified *Nc*CDH and the tested sugars. The spectra show is averaged over a retention time (RT) approx. +1 to -1 minute before and after the respective oxidized sugar eluted in the chromatogram. Oxidized sugar were detected for D) maltose, (7.9 min,381), E) lactose (7.7 min, 381), F) cellobiose (8.5 min, 381), and G) maltotriose (9.2 min, m/z 543). For the control no time was selected and the full scan is displayed.

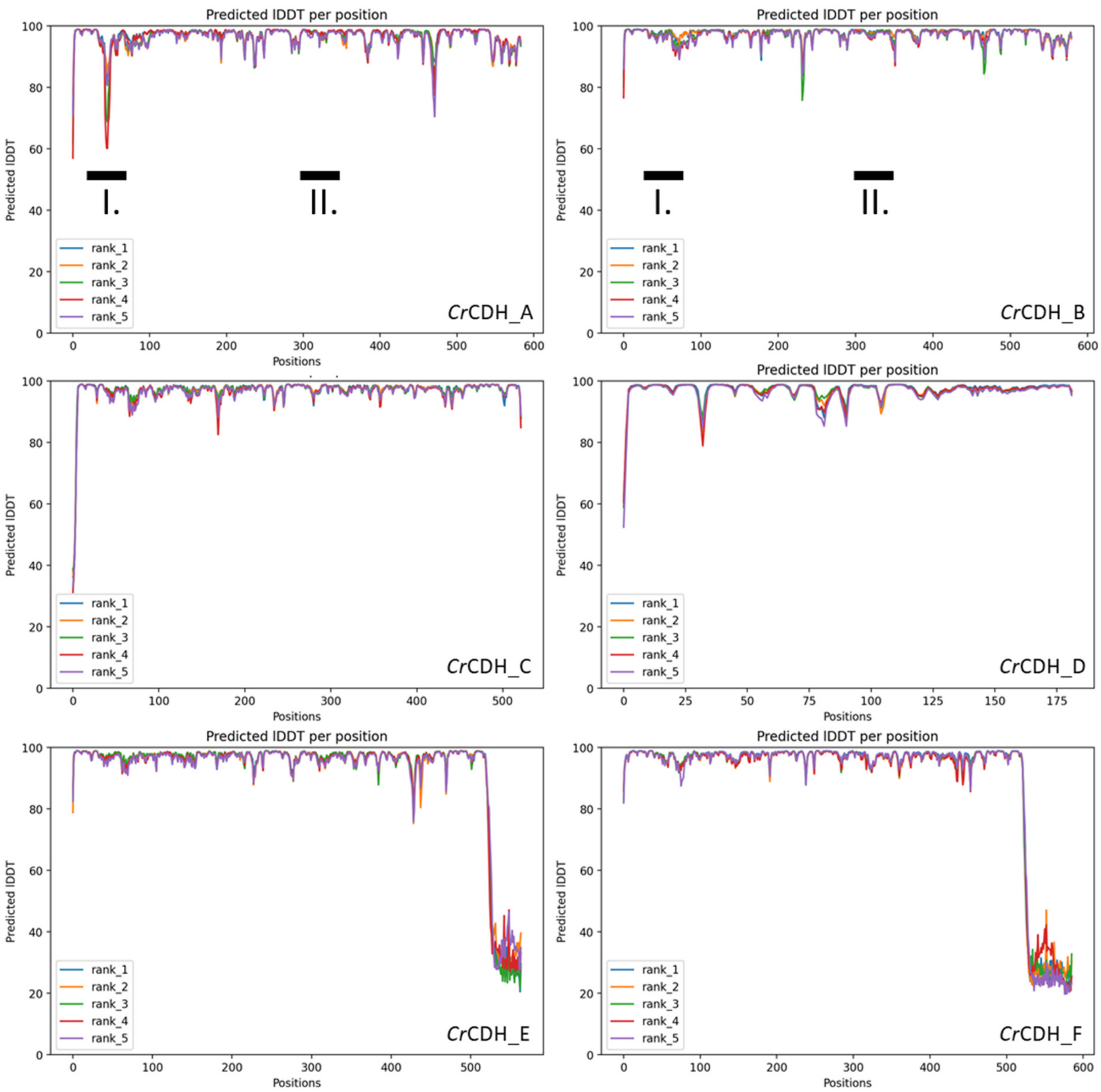

Supplementary Figure 19: Predicted local structural confidence (pLDDT) along the DH domain of the protein sequences for *Cr*CDH_A to F. Per-residue pLDDT values are shown for the top five AlphaFold models (rank_1 to rank_5). The black lines indicate the loops around the active site with high RMDS values in *Cr*CDH_A and *Cr*CDH_B compared to their templates. Regions I. and II. represent the loops at amino acid positions 72 to 92, and 325 to 355 in the DH domain of *Cr*CDH_A, respectively. The x-axis represents residue position along the input sequence, and the y-axis indicates predicted structural confidence on a 0–100 scale, where higher values reflect greater confidence.
